# Supplementary material for: Deep CRISPR mutagenesis characterizes the functional diversity of TP53 mutations
Source: Nat Genet. 2025 Jan 7;57(1):140–53. doi: 10.1038/s41588-024-02039-4 (PMC11735402; doi:10.1038/s41588-024-02039-4)
Supplement: Supplementary file 1 — Supplementary Notes 1–7, Discussion, Methods 1–10, Figs. 1–12 and References. [file 41588_2024_2039_MOESM1_ESM.pdf]

nature genetics

Supplementary information

https://doi.org/10.1038/s41588-024-02039-4

Deep CRISPR mutagenesis characterizes the functional diversity of *TP53* mutations

In the format provided by the authors and unedited

Table of Contents

Table of Contents **1**

Supplementary Notes **2**

Supplementary Note 1: Isogenic model for *TP53* mutagenesis by CRISPR-HDR 2
Supplementary Note 2: Single-cell RNA-seq analysis for clonal variability 3
Supplementary Note 3: Pro-metastatic GOF analysis for HCT116 R175H cells 4
Supplementary Note 4: Validation in H460 cell line 5
Supplementary Note 5: Correlation of RFS and mutational probabilities 6
Supplementary Note 6: Detailed description of variants causing splice alterations 7

Supplementary Discussion **9**

Supplementary Methods **13**

Supplementary Method 1: Cell Culture 13
Supplementary Method 2: Reverse transcription quantitative PCR (RTqPCR) 13
Supplementary Method 3: RNA sequencing 14
Supplementary Method 4: Single-cell RNA sequencing 14
Supplementary Method 5: Western blot analysis 15
Supplementary Method 6: Stability measurements of recombinant p53 variants 15
Supplementary Method 7: Proliferation/IC_{50} assay 16
Supplementary Method 8: Apoptosis assay 16
Supplementary Method 9: Invasion/migration assays 16
Supplementary Method 10: Animal experiments 17

Supplementary Figures **19**

Supplementary Figure 1: p53 target gene regulation in edited HCT116 cells 19
Supplementary Figure 2: R175 variants and Mdm2/Mdmx inhibitor responses 20
Supplementary Figure 3: Mutant p53 reactivation by APR246 and ZMC1 21
Supplementary Figure 4: Separation-of-function phenotype of R175 variants 22
Supplementary Figure 5: DBD variant coverage in *TP53* DMS studies 23
Supplementary Figure 6: CRISPR RFS scores versus transcriptional activity 24
Supplementary Figure 7: CRISPR versus cDNA overexpression screen 25
Supplementary Figure 8: Temperature-sensitive function of LR variants 26
Supplementary Figure 9: Fitness impact of variants near exon-intron borders 27
Supplementary Figure 10: Gating strategy for apoptosis analysis 28
Supplementary Figure 11: Unprocessed Blots for Supplementary Figure 4c 29
Supplementary Figure 12: Unprocessed Blots for Supplementary Figure 8a 30

Supplementary Information References **31**

Supplementary Notes

Supplementary Note 1: Isogenic model for *TP53* mutagenesis by CRISPR-HDR

In order to compare the functional impact of *TP53* variants in an isogenic setting, we chose HCT116 colorectal carcinoma cells as our model system. These cells are wild-type for *TP53* and show a prototypical p53 response, making them a well-established model for investigating the mechanistic details of p53-mediated tumor suppression[1-4]. To ensure unambiguous genotype-phenotype correlations, we haploidized the cells for *TP53* by deleting intronic splicing branch points in one of the two *TP53* alleles (Δ allele, Fig. 1a and Extended Data Fig. 1a-c). To prevent potential bias from the anti-proliferative DNA damage response triggered by p53 in response to CRISPR/Cas9-induced DNA strand breaks[5-7], we refined existing technologies for saturation genome editing[8-10]. Specifically, we reversibly silenced expression from the remaining copy by inserting a LoxP-flanked transcriptional stop cassette (Lox-Stop-Lox, LSL) into intron 4 (HCT116 LSL/Δ). The LSL cassette included an EGFP expression cassette for monitoring and a mutated puromycin N-acetyltransferase (*pac*) gene that could be repaired during editing for later selection. The LSL allele was specifically cleaved by CRISPR/Cas9 nucleases, targeting the intronic regions deleted on the Δ allele, to stimulate repair of the introduced DNA double-strand break via the homology-directed repair (HDR) pathway. Specific mutations of interest were introduced into the cleaved LSL allele through HDR, using co-transfected donor vectors that serve as repair templates. These donor vectors contain the desired mutations, a mutated protospacer adjacent motif (PAM) to prevent re-cutting, and the LSL-cassette with a repaired *pac* gene. Correctly HDR-edited cells (HCT116 LSL-mut/Δ) were selected with puromycin and infected with Cre recombinase expressing adenovirus (AV-Cre) to induce monoallelic p53 variant expression (HCT116 mut/Δ).

We validated the performance of *TP53* editing in HCT116 LSL/Δ cells by introducing a panel of *TP53* variants, including some of the most frequent cancer mutations (V157F, R175H, C176F, H179R, Y220C, G245S, R248Q, R248W, R249S, R273H) with known LOF, the partial LOF (pLOF) mutations R175P and R181L, the nonsense mutation R175X and the wild type (WT) for reference. Single-cell clones were analyzed by PCR, revealing donor integration in 75.9% of cases (Fig. 1b). Since homologous recombination can occur anywhere within the homology arms, we further assessed the presence of the specific mutations by sequencing, which showed a 56.4% success rate (Fig. 1b). Excision of the LSL cassette with AV-Cre resulted in comparable protein expression levels of WT and all p53 missense mutants, which was further enhanced by inhibition of Mdm2 with Nutlin-3a (N3a) (Fig. 1c and Extended Data Fig. 1d). Consistent with the expected LOF of many cancer mutants, N3a induced expression of p21/CDKN1A protein and characteristic p53 gene expression signatures only in WT and R181L mutant cells (Fig. 1c-e, Supplementary Fig. 1). Real-time live-cell imaging of N3a-treated cells demonstrated a strong reduction in proliferative fitness in WT cells, which was diminished by pLOF mutations and completely abrogated by LOF missense and nonsense mutants (Fig. 1f, g, Extended Data Fig. 1f).

Supplementary Note 2: Single-cell RNA-seq analysis for clonal variability

Notably, different single-cell clones exhibited some phenotypic variation, prompting the question of whether these are clonal artifacts or true phenotypic differences potentially caused by variant-specific GOF effects (Fig. 1d, Extended Data Fig. 1e, g, Supplementary Fig. 1). To investigate this issue, we analyzed the transcriptome of a cell pool composed of 12 different *TP53* variants (8 missense, 3 nonsense and wild type), with each variant represented by 10 independent single-cell clones, using single-cell RNA sequencing (Extended Data Fig. 2). Dimensionality reduction analysis, employed to visualize the overall distribution of N3a- and DMSO-treated cell populations, delineated two main cell clusters. Cluster 1, marked by high-level expression of p53 target genes, predominantly contained N3a-treated WT clones, while cluster 2, enriched in cell-cycle genes, encompassed DMSO-treated WT clones alongside clones harboring all missense and nonsense variants, regardless of treatment. Within cluster 2, cells separated based on cell-cycle phase rather than by *TP53* genotype or clonal identity. The distinct clustering of N3a-treated WT clones apart from a uniform cluster comprising all other clones confirms the anticipated LOF effect of the analyzed missense and nonsense variants, demonstrates minimal intercellular heterogeneity and clonal variance, and argues against mutant-specific GOF effects in this experimental setting.

Supplementary Note 3: Pro-metastatic GOF analysis for HCT116 R175H cells

The GOF typically depends on secondary alterations in transformed cells that constitutively stabilize the mutant p53 protein, which is inherently unstable in non-transformed cells[11-14]. Constitutive stabilization was not observed for the engineered missense mutants in HCT116 cells. All missense mutants were expressed at levels comparable to wild-type p53 in parental HCT116 and other non-transformed cell types (Fig. 1c, Extended Data Fig. 1h). N3a stabilized the mutant p53 proteins, but the levels remained significantly lower than in cell lines derived from patient tumors with naturally acquired *TP53* mutations (Fig. 1c, Extended Data Fig. 1d, e, i-j).

The GOF of missense variants, particularly R175H, is best documented for metastasis-promoting properties[15,16]. Therefore, we compared the migratory behavior of HCT116 cells with the R175H missense and the R175X nonsense mutations using transwell assays. However, the migration of both cell types was indistinguishable, even in the presence of N3a (Extended Data Fig. 3a-c). Additionally, both cell types were intravenously injected into mice, and after several weeks, tumors were dissected from lungs and metastatic sites such as the liver. The explanted tumor cells were expanded *ex vivo* and re-injected into mice for a total of three passages (Extended Data Fig. 3d, e). Over months of serial *in vivo* propagation, we observed a progressive increase in migratory and invasive behavior for R175H cells, which was not explained by differences in proliferation and was not observed with the R175X nonsense mutation (Extended Data Fig. 3f-k). Migration and invasion, but not proliferation, of late passage R175H cells were significantly reduced by knock-down or knock-out of the mutant (Extended Data Fig. 3h-o). Furthermore, knock-out of the mutant in late passage R175H cells did not affect the growth of subcutaneously grown tumors but significantly impaired metastasis to the liver (Extended Data Fig. 3p-r). Intriguingly, baseline protein levels of R175H increased progressively with serial *in vivo* passaging (Extended Data Fig. 3e), suggesting that the enhanced metastatic properties are driven by the selection of cells that have acquired mechanisms to constitutively stabilize the mutant protein.

Supplementary Note 4: Validation in H460 cell line

To examine the cell type-specificity of the results, we performed a mutagenesis scan in the non-small cell lung cancer cell line H460, which carries three wild-type copies of *TP53*. Analogously to HCT116 LSL/Δ cells, we engineered the cells to enable conditional expression of one wild-type allele and deleted the remaining two copies, resulting in H460 LSL/Δ/Δ cells (Extended Data Fig. 4a, b). After confirming the efficacy of the editing process (Extended Data Fig. 4c, d), we introduced the R175 variant library into the editable LSL allele, activated expression with Cre, and measured the response of the H460 mut/Δ/Δ cell library to N3a treatment as described for HCT116 cells (Extended Data Fig. 4e, f). The N3a-induced changes in the abundance of individual p53 variants were significantly correlated between H460 cells and HCT116 cells (ρ=0.969, p<0.0001), indicating that the fitness effect of mutations is highly conserved across different cell types.

Supplementary Note 5: Correlation of RFS and mutational probabilities

We observed a high number of missense mutations at evolutionary conserved residues that scored high RFS values but were never or rarely reported in patients (Fig. 5b, c), raising the question whether these are false positives of our screen. Many of these variants were either 2 or 3 nt substitutions or single-nucleotide transversions (Extended Data Fig. 6b, c) that are all less frequent in cancer cells than single-nucleotide transitions[17]. While most of the hotspot mutations affect CpGs, none of the never observed high-RFS variants were at CpG sites (Extended Data Fig. 6d). Nevertheless, irrespective of the type of mutation, variants with a positive RFS had significantly higher patient counts than those with negative RFS (Extended Data Fig. 6e-h), suggesting that they are selected for during tumorigenesis.

We therefore also evaluated the sequence context of the screened variants for their mutational probability based on COSMIC mutational signatures that represent the major mutational processes accounting for base substitutions in cancer genomes (Extended Data Fig. 6i-l, Supplementary Table 5)[18]. Variants with positive RFS and high patient counts showed much higher mutational probabilities for all relevant mutational processes than high-RFS variants with low or zero patient counts (Extended Data Fig. 6i). In addition, variants with negative RFS despite high mutational probability have an average patient count below 100, underlining that these are random passenger mutations which are observed repeatedly in cancer patients only because of their high mutational probability, not because they disrupt p53 function. When comparing variants with similar mutational probabilities, those with a positive RFS consistently showed significantly higher patient counts than those with a negative RFS (Extended Data Fig. 6k and l). A positive RFS therefore robustly identifies loss-of-function variants, which are under positive selection during tumor development.

Supplementary Note 6: Detailed description of variants causing splice alterations

Interestingly, several missense mutations were also underrepresented at the mRNA level and associated with an LOF (Extended Data Fig. 9a). Most of these variants are located close to exon/intron borders (Extended Data Fig. 9b), supporting the hypothesis that they might affect mRNA splicing. While many are rare double or triple nucleotide substitutions, some are caused by single nucleotide substitutions (SNVs) and have been reported in several cancer patients (Extended Data Fig. 9c, d). Of particular significance are variants at codons G187, E224, and S261, which are prevalent in cancer samples and have been classified as WT-like in all cDNA overexpression screens[19-21]. To validate the LOF predicted by our CRISPR-based DMS, we introduced into HCT116 LSL/Δ cells the variants NC_000017.11:g.7674859C>G, encoding the putative missense mutant E224D, and NC_000017.11:g.7674859C>T, a presumptive silent variant E224=, which affect the last nucleotide of exon 5 and have been reported in 77 and 24 patients, respectively. Sequencing of cDNA revealed that both variants attenuate the wild-type 5' splice site (5' ss) of intron 6 and enforce the use of an aberrant downstream 5' ss (Fig. 7f). The resulting inclusion of 5 bp from intron 6 into the mature mRNA causes a frameshift with a premature termination codon (PTC) in an alternative reading frame of exon 7. Consequently, the mRNA is subject to NMD, which prevents the production of a truncated protein and renders cells resistant to N3a (Fig. 7g-i).

Away from exon/intron borders, we also observed LOF variants in the non-coding, exon-flanking, intronic regions that could be attributed to altered splicing. For example, we expectedly observed an LOF for most essential splice site mutations, i.e. for substitutions affecting the nearly invariant GT and AG dinucleotides at the intron ends (Supplementary Fig. 9). Moreover, we detected a deleterious impact of all SNVs at position 5 of intron 5, consistent with a strong prevalence for G at this position in the 5' ss consensus sequence. While the impact of these mutations would have been predicted based on consensus sequences, introns 6-8 also harbor a G at this conserved position but are tolerant to all substitutions. Consistently, the GENIE database lists 10 cases of various G5 substitutions for intron 5, but only a single case for introns 6-8. We also noted an LOF associated with the single nucleotide substitution NC_000017.11:g.7673847A>C in the 3' region of intron 7 (Supplementary Fig. 9), which has been reported by the TCGA in a pancreatic adenocarcinoma patient[22]. Consistent with the creation of a cryptic 3' ss, we observed an aberrant inclusion of 9 bp of intronic sequence causing an in-frame insertion of 3 amino acids (Extended Data Fig. 9e, f). Different from cDNA-based screens, the CRISPR screen therefore discriminates functionally normal from abnormal variants even in non-coding intronic regions.

Interestingly, we also noted reduced mRNA levels for the exonic codon 137 variant NC_000017.11:g.7675202A>T, encoding the putative missense variant L137Q (Extended Data Fig. 9). In addition, we observed reduced mRNA levels of the variant NC_000017.11:g.7674934T>A, encoding the putative synonymous variant G199=, while two other predicted silent substitutions at this position, NC_000017.11:g.7674934T>G/C, were present at the expected mRNA level (Extended Data Fig. 9). Although the transversion g.7674934T>A at codon G199 has a low mutational probability and has not yet been reported in a cancer patient, the codon L137 variant g.7675202A>T has been recurrently found in cancer patients. Interestingly, none of these variants has shown an LOF in any of the cDNA

overexpression screens. Nevertheless, closer inspection predicted the creation of a cryptic 5' ss within exon 6 by g.7674934T>A and a new 3' ss in exon 5 by g.7675202A>T. To validate the potential LOF due to an impact on mRNA splicing, we separately introduced them into both HCT116 and H460 cells and characterized their response to N3a (Fig. 8 and Extended Data Fig. 10).

Confirming the CRISPR-based DMS results, g.7674934T>A cells lacked an anti-proliferative N3a-response (Fig. 8a-c and Extended Data Fig. 10a-c). They expressed little to no p53 protein and failed to induce p21 when treated with N3a (Fig. 8d and Extended Data Fig. 10d). Notably, g.7674934T>G/C cells showed a WT-like phenotype (Fig. 8a-d and Extended Data Fig. 10a-d). Sequencing of the cDNA from g.7674934T>A cells revealed a complex, previously unpredicted, pattern of transcripts, dominated by transcripts either lacking exon 6 completely or containing a 3'-truncated exon 6 with or without inclusion of intron 5 (Fig. 8e, f and Extended Data Fig. 10e, f). All these aberrant transcripts contained premature stop codons, explaining the observed lack of p53 protein.

In line with the CRISPR-based DMS results, cells harboring the codon L137 variant g.7675202A>T also lacked an anti-proliferative N3a-response and failed to induce p21, indistinguishably from cells with the R175H missense or R175X nonsense mutation (Fig. 8a-d and Extended Data Fig. 10a-d). g.7675202A>T cells expressed a shorter p53 protein, and cDNA sequencing revealed a single aberrantly shortened transcript containing a 5' truncated exon 5 (Fig. 8g-i and Extended Data Fig. 10g-i). This aberrantly spliced mRNA generates a shortened protein with a deleterious in-frame deletion of amino acids 126-137 and prevents the production of a full-length L137Q protein, which was analyzed in cDNA overexpression screens and classified as WT-like. As splice alterations can be reverted with splice-switching oligonucleotides (SSO)[23], we transfected g.7675202A>T cells with an SSO specifically designed to block the cryptic 3' ss created by the variant (Fig. 8j and Extended Data Fig. 10j). The SSO transfection significantly increased the regularly-spliced p53 mRNA level and sensitized to p21 induction by N3a. This confirms the LOF of the g.7675202A>T variant to be due to aberrant splicing, rather than a nonfunctional L137Q protein, and also provides proof-of-principle evidence that a cancer-associated p53 splice aberration can be corrected using SSO technology. However, g.7675202A>T and g.7674934T>A were the only SNVs not located at exon/intron borders that showed a more than 2-fold reduction of the expected full-length mRNA and resulted in an LOF (Extended Data Fig. 9a), although 355 missense or synonymous SNVs create new exonic GT or AG dinucleotides that could theoretically function as cryptic splice sites. Splice aberrations caused by intra-exonic SNVs are therefore far less frequent than anticipated.

Supplementary Discussion

The comprehensive deep mutational scan using saturation genome editing by CRISPR-HDR reported in this paper covers approximately 94.5% of all cancer-associated *TP53* mutations. This study should be viewed in the context of previous MAVE studies on *TP53* using overexpression of mutant cDNA[19-21]. While cDNA-based MAVE studies failed to cleanly separate nonsense from synonymous and known pathogenic from known benign variants[24], the CRISPR approach showed superior separation not only for WT-like and LOF variants but also for pathogenic and benign ClinVar variants (Fig. 6a, Extended Data Fig. 7). An arbitrary RFS cut-off of zero demonstrated predictive values, sensitivity and specificity exceeding that of classifiers based on a combination of four cDNA-based MAVE datasets[24], and provided a strength of evidence for variant interpretation reaching the strong PS3 and BS3 levels according to ACMG/AMP guidelines (Supplementary Table 6)[25-27]. Despite the fact that lentiviral integration is not entirely random, the increased variability in mutant expression levels in the cDNA overexpression screens may be explained by highly variable lentiviral integration, occurring at differently active genomic regions[28]. In contrast, CRISPR introduces the mutation into a defined genomic locus, here the endogenous *TP53* gene, resulting in highly consistent and physiological protein expression and reproducible transcriptomic and fitness effects between independently edited clones (Extended Data Fig. 1e, g and 2).

As a result of the observed improvement in discriminatory power, we identified approximately 20% of the missense variants as fitness-promoting mutations that were missed by cDNA-screening (Fig. 6b). A closer examination showed that these variants have a similar mutational probability but are significantly more frequent in tumors compared to synonymous or WT-like variants (Fig. 6c, d), indicating that they are dysfunctional variants selected during tumorigenesis, which is further demonstrated by the tumorigenicity of some of these variants in mice[29-31]. This suggests that the deleterious impact of many *TP53* variants has been underestimated previously due to technical limitations that can be overcome by a CRISPR screening approach.

Interestingly, many of these variants were predicted to be thermally destabilized by only a few degrees of temperature (Fig. 6i), significantly less than some of the more frequent structural hotspot mutants such as Y220C[32,33]. This could explain their overall higher residual transcriptional activity (Fig. 6g, h) and their lower frequency in cancer patients (Fig. 6d), but also identifies them as potentially temperature-sensitive, which was confirmed by a detailed analysis of representative mutations (V157L and T256A, Supplementary Fig. 8). From a translational perspective this suggests that the folded, active conformation of these mutants might be more easily rescued[34,35], making these variants an especially interesting subset for pharmacological targeting approaches (Supplementary Table 8). It is tempting to speculate that cancer patients with variants from this subset might benefit most from therapeutic approaches suggested for temperature-sensitive mutations, such as hypothermia and antiparasitic antimonials[36,37].

Large-scale RNA sequencing studies of cancer samples have uncovered several examples of splice alterations attributed to intronic and exonic missense and synonymous variants within crucial cancer genes like *TP53*[38-41]. These findings underscore the imperative for a more

systematic experimental exploration of variant effects on splicing. While cDNA-based screens are inherently unable to detect splice alterations[39], the unbiased CRISPR-based mutagenesis of the endogenous *TP53* locus allowed us to comprehensively delineate splice alterations with fitness-promoting effects. As expected from previous studies[38-40], substitution variants that affect regular splicing tend to cluster at exon/intron borders, either by altering the GT and AG dinucleotides (5' and 3' ss) at the intron ends or by changing the first or last exonic nucleotides, where mutations can weaken the regular splice sites and promote the usage of alternative ones[42]. In addition, we also observed two exonic SNVs with splicing effects (g.7675202A>T and g.7674934T>A), which were both classified by cDNA overexpression screens as benign missense (L137Q) and synonymous (G199=) variants, respectively. Our screen and validation analysis in HCT116 and H460 cells revealed that both SNVs create cryptic splice sites, resulting in aberrantly spliced transcripts that enhance tumor cell fitness (Fig. 8 and Extended Data Fig. 10). The g.7675202A>T variant at codon 137 induces a new 3' splice site, leading to a smaller protein with an in-frame deletion of 12 amino acids. Conversely, the g.7674934T>A variant at codon 199 generates a complex pattern of aberrantly spliced transcripts with premature stop codons, triggering NMD and preventing p53 protein production. Notably, these were the only two SNVs outside the exon/intron border regions of exons 5-8 that modified splicing patterns. This is especially noteworthy because many more SNVs create potential cryptic splice sites without disrupting regular splicing or impairing p53 function. In addition, it was anticipated that SNVs located at putative exonic splicing enhancers/silencers would have splicing effects. However, the lack of splice alterations by most exonic SNVs suggests that *TP53* splicing in the DBD region is overall robust and that SNVs in this critical region typically fail to establish functional splice sites or interfere with splicing enhancers/silencers. However, SNVs that create a novel splice site are removed from the mRNA during splicing, so we can only infer their splicing effect indirectly from the absence of a normally spliced mRNA encoding the variant. It is therefore possible that some SNVs produce a mixture of normal and additional, aberrantly spliced transcripts. A comprehensive analysis integrating DNA and mRNA from variant libraries at the single-cell level is necessary to fully understand the extent and functional impact of such variants.

Our study primarily used Mdm2 inhibition with N3a as a specific method to activate p53. This approach is based on the understanding that p53 is ultimately activated by disrupting the p53-Mdm2 interaction, regardless of the stress factor involved. As such, we observed a similar variant enrichment/depletion pattern with various p53-activating stress factors, including DNA damage and metabolic stress, compared to N3a or other Mdm2/Mdmx inhibitors (Fig. 3a, b). However, the level of enrichment/depletion was less pronounced with other stressors as additional stress pathways unrelated to p53 also affect tumor cell fitness.

It is widely believed that p53 is more frequently hit by missense mutations at mutational hotspot codons compared with other tumor suppressors because they can provide the mutant protein with pro-tumorigenic GOF properties, which in many cases rely on the modulation of signaling pathways through interactions of mutant p53 with other transcription factors or chromatin remodellers[11,43]. However, this notion is somewhat challenged as transcriptional signatures easily distinguish wild-type tumors from tumors with *TP53* mutations, while separation of tumors with missense from nonsense mutations is difficult to impossible[44,45]. Moreover, the unique *TP53* mutation spectrum of cancer patients can be computationally

modeled fairly accurately without requiring the consideration of GOF effects[21]. Together, this suggests that the loss of wild-type function (LOF) is the primary driver of tumor initiation, while GOF activities may contribute to tumor progression only at later stages. In support of this, GOF effects usually require constitutive stabilization of mutant p53 proteins caused by secondary genetic alterations such as aneuploidy, *TP53* LOH, and loss of p16INK4A or Mdm2[12,46,47]. Despite using transformed cells derived from colorectal (HCT116) or lung cancer (H460), the CRISPR-engineered missense mutations did not result in the constitutive stabilization of mutant p53 in our studies. Mutant p53 protein expression remained at the level of wild-type p53 and under Mdm2 control (Fig. 1c, 8d and Extended Data Fig. 1d, e, h-j, 4d, 10d, Supplementary Fig. 8). This supports the notion that p53 missense mutants are inherently unstable not only in non-transformed cells but also in fully transformed cells, unless specific alterations stabilize the mutant protein. Consistent with this concept, we observed progressively increasing R175H expression levels along with a gain of pro-metastatic traits in R175H-mutant HCT116 cells after prolonged cell passaging in mice (Extended Data Fig. 3). These enhanced metastatic traits remained dependent on sustained R175H expression and seemed to result from a long-term selection process, rather than acute stabilization of R175H with N3a. Recent genome-wide CRISPR knock-out screens have delineated pathways and networks of factors that control mutant p53 stability[48]. It will be intriguing to analyze how these networks change during tumor evolution to stabilize mutant p53 and unleash pro-tumorigenic GOF effects. The absence of constitutive mutant p53 stabilization in our multiplexed assay for fitness effects enabled us to specifically capture the LOF effects, which are considered most critical for the tumorigenicity of a mutation, in the absence of confounding GOF effects. To interrogate GOF properties, a mutational scan in a cell line harboring constitutively stabilized mutant p53 could be considered, but might face gene editing challenges due to the genetic instability and aneuploidy of most p53-mutated cells. Moreover, as GOF effects are highly diverse and extend far beyond proliferation effects, identification of read-outs suitable for high-throughput screening will be a prerequisite.

Limitations

The study was limited in its ability to examine the dominant-negative activity of variants that promotes tumorigenesis in the presence of a remaining wild-type allele[49,50]. Our HCT116 LSL/Δ screening cell line has an inactive second allele. To study dominant-negative effects, we attempted to generate an LSL/WT cell line, but gene editing in these cells was less efficient, potentially due to wild-type p53 interference with HDR-mediated DSB repair[5-7]. Additionally, these cells were genetically unstable and, like other p53+/- cells[51,52], underwent LOH at high frequency. We also confirmed our results in a different cellular context, the p53 wild-type, non-small cell lung cancer cell line H460. We successfully introduced single mutations and a small R175 library into this cell line using the same approach as in HCT116 (Extended Data Fig. 4). The results were similar to those with HCT116 cells, indicating comparable LOF effects of variants regardless of the cell type. However, the transfection and editing efficiency were inadequate for larger screens. HCT116 cells have already been widely used in the pre-CRISPR era for mutagenesis by homology-directed repair due to their mismatch repair deficiency[1,53,54], and dominant-negative inhibition of MMR has also been shown to improve gene editing with prime editors[55]. It is possible that the exceptional HDR efficiency we observed in our screen

was facilitated by this, and that transient inhibition of MMR might be a strategy to enable similar screens in other model systems.

Supplementary Methods

Supplementary Method 1: Cell Culture

The human colorectal carcinoma cell line HCT116, the human large-cell lung cancer cell line NCI-H460 (H460), human embryonic kidney cell line HEK 293T, mammary epithelial cells (MCF10A) and human cell lines from *TP53*-mutated tumors (LS-123, MIA-PaCa2, PC-9, NCI-H1975 (H1975), PANC-1) were obtained from the American Tissue Collection Center (ATCC, HCT116: CCL-247, H460: HTB-177, HEK293T: CRL-3216, MCF10A: CRL-10317, LS-123: CCL-255, MIA-PaCa2: CRL-1420, H1975: CRL-5908, PANC-1: CRL-1469) or from the European Collection of Authenticated Cell Cultures (ECACC, PC9: 90071810). Normal human diploid fibroblasts (NHDF) and normal human epidermal keratinocytes were obtained from healthy donors. Cell lines were cultured in Dulbecco's modified Eagle's medium (DMEM, Gibco, 41966-029, used for HCT116, HEK293T, NHDF, LS-123, MIA-PaCa2 and PANC-1) or Roswell Park Memorial Institute (RPMI) 1640 medium (Gibco, 61870, used for H460, PC-9, and H1975) supplemented with 10% heat-inactivated fetal bovine serum (FBS, Sigma-Aldrich, S0615) and 100 U/ml penicillin and 100 µg/ml streptomycin (Gibco, 15140122). Human Mammary Epithelial Cell Growth Medium (Merck, 815-500) and Keratinocyte Growth Medium 3 (PromoCell, C-20021) were used for MCF10A and NHEK, respectively. Cell lines were cultivated in a humidified atmosphere at 37 °C and 5% CO_2 and detached using 0.05% Trypsin-EDTA (Gibco, 15400054) or the Detach Kit (PromoCell, C-41200, used for NHEK, MCF10A) according to the manufacturer's protocol. HCT116 were authenticated by genome sequencing before and multiple times during the course of the experiments. NCI-H460 were authenticated by STR profiling, LS-123, MIA-PaCa2, H1975, PANC-1, PC9, and MCF10A by profiling their *TP53* mutation status before the start of the experiments. HEK 293T and AmphoPack-293 were authenticated functionally by their lentiviral packaging capacity.

Transfection of cells with splice-switching oligonucleotides. Antisense oligonucleotides with complete 2'MOE modifications (ASO 2'MOE) were purchased from Integrated DNA Technologies. Cells were electroporated with 250 nM of the ASO: 5'-GTC TTG GCC TGT TGG CAA AAC A-3' using the Neon Transfection System (Thermo Fisher Scientific, MPK10025) according to the manufacturer's protocol. To account for potential off-target effects, data from g.7675202A>T cells were normalized to R175X cells transfected and treated in parallel using the same procedures.

Supplementary Method 2: Reverse transcription quantitative PCR (RTqPCR)

Total RNA was isolated using the RNeasy Mini Kit and reverse transcribed using the SuperScript VILO cDNA Synthesis Kit. The resulting cDNA was used for qPCR using ABsolute qPCR Mix SYBR Green (Thermo Fisher, AB1158B) with the following primers: TP53_RTqPCR_ fw/rev, CDKN1A_RTqPCR_fw/rev, GAPDH_RTqPCR_fw/rev. Quantitative PCR was performed using the LightCycler 480 (Roche Diagnostics) and the LightCycler 480 software (v1.5.0.39). The regularly spliced *TP53* transcript in cells with the

g.7675202A>T variant was quantified with the following primers: TP53_L137Q_fw/rev. The data were analyzed using the $\Delta\Delta C_t$ method with GAPDH as reference gene.

Supplementary Method 3: RNA sequencing

For RNA sequencing experiments, cells were treated either with 10 µM N3a or the corresponding volume of DMSO, as solvent control, for 36 h prior to RNA isolation using the RNeasy Mini Kit according to the manufacturer's protocol. RNA quality was evaluated using the Experion RNA StdSens Analysis Kit (Bio-Rad, 700-7103). RNAseq libraries were prepared from total RNA with the QuantSeq 3′ mRNA-Seq Library Prep Kit FWD for Illumina (Lexogen, 015.24) in combination with the UMI Second Strand Synthesis Module for QuantSeq FWD (Illumina, Read 1) (Lexogen, 081.96) following the manufacturer's protocol. The quality of sequencing libraries was validated on a Bioanalyzer 2100 using the Agilent High Sensitivity DNA Kit. Pooled sequencing libraries were quantified and sequenced on the NextSeq 550 platform (Illumina) with 75-base single reads. Unique molecular identifiers (UMI) were extracted and the first four nucleotides corresponding to the QuantSeq FWD-UMI 3' spacer were removed. The trimmed reads were mapped to the Homo sapiens (revision 104, GRCh38) Ensembl reference genome using STAR[56] (v2.7.10a). The UMIs were then deduplicated using UMI-tools[57] (v1.1.1). The UMI per gene was quantified and normalized to counts per million (CPM). Genes with CPM counts below 1 in all samples were considered background noise and discarded. Further analysis was restricted to protein-coding and lincRNA genes. Differential gene expression was analyzed via DEseq2 (v1.34.0)[58]. The obtained p-values were corrected via Benjamini-Hochberg correction. Genes with log2FC ≥ 1 as well as corrected p-values smaller than 0.05 were considered differentially expressed. Principle component analysis was performed on the corrected count matrix after subjecting it to variance-stabilizing transformation via DEseq2. For heatmaps, expression values were z-transformed and genes were clustered using hierarchical average-linkage clustering based on Euclidean distances. Gene set enrichment analysis was performed using gene sets from the Molecular Signatures Database (MSigDB) and GSEA software[59] (v4.2.2).

Supplementary Method 4: Single-cell RNA sequencing

Ten individually generated single-cell clones of the indicated HCT116 mut/Δ cell lines were labeled by lentiviral transduction with a specific combination of expressed SPLINTR barcodes (gift from Mark Dawson, Addgene #179774 and #179775)[60]. *TP53* variants were encoded by GFP-coupled barcodes and clone IDs by mCherry-coupled barcodes. All barcoded cell clones were pooled and split into two aliquots. One aliquot was treated with 10 µM N3a, the other with the corresponding volume DMSO for 24 h. Single-cell RNA sequencing libraries were prepared from both pools (50,000 cells in total) using the Evercode WT v2 kit (Parse Biosciences) according to the manufacturer's protocol. Quality of sequencing libraries was controlled on a Bioanalyzer using the Agilent High Sensitivity DNA Kit (Agilent) and quantified with the Qubit dsDNA-HS-Assay-Kit (Thermo Fisher Scientific). Libraries were pooled and sequenced on a NovaSeq 6000 S2 flow cell (Illumina) with paired-end reads (114 bases read1, 86 bases read2).

Reads from different libraries were combined using the ParseBiosciences pipeline (v1.1.0) using the "comb" option with default parametrization. Barcode combinations that identify

single cells were extracted from the R2 reads, corrected for sequencing errors, and used to tag R1 cDNA reads. Reads were then aligned against the Ensembl Homo sapiens reference genome (revision 109, GRCh38) using STAR (v2.7.10a). Gene/transcript counts were inferred from the alignments and subjected to further analysis using scanpy (v1.9.0). Read counts were normalized to a total count of 1e6 reads, logarithmized and scaled to center around zero and exported as a DGE matrix via the ParseBiociences pipeline. To infer the actual *TP53* genotype per cell, the reads were additionally aligned via STAR to a custom genome comprising all of the SPLINTR barcodes used to label the cells. A cell was annotated with a distinct *TP53* genotype if a unique mapping against exactly one GFP-SPLINTR barcode was found for reads obtained from that specific cell. For further analysis, the DGE matrix was filtered to include only cells that contained at least counts for 2,000 genes, and genes were filtered to those that were detected in at least 10 cells. In total, 40,191 cells remained that also passed quality control. The percentage of mitochondrial and ribosomal reads per cell was calculated and the DGE matrix was corrected by regressing out the total counts as well as mitochondrial and ribosomal read percentages. In addition, the gene counts were regressed out. Subsequently, principal component analysis was performed, followed by calculation of a neighborhood graph (n_neighbors=50, n_pcs=50), leiden clustering, and partition-based graph abstraction (PAGA). The PAGA graph was then used to initialize UMAP calculation. In order to score the *TP53* pathway activation, genes from the HALLMARK_P53_PATHWAY gene signature were used (obtained from MSigDB, v2023.2.Hs).

Supplementary Method 5: Western blot analysis

Cells were lysed in NP-40 Lysis Buffer (50 mM Tris-HCl, 150 mM NaCl, 5 mM EDTA, 2% NP-40, pH 8.0) supplemented with cOmplete ULTRA protease inhibitor cocktail (Roche, 4693124001) and sonicated using a Bioruptor (Diagenode) for 5 x 30 seconds. 20-40 µg of protein was separated on NuPAGE 4 to 12% Bis-Tris polyacrylamide gels (Invitrogen, WG1402) using MOPS buffer (Invitrogen, NP0001). Following transfer to Immun-Blot PVDF Membrane (BioRad, 1620177), antigens were detected using the antibodies: p53 (Santa Cruz Biotechnology, sc-126; antibody DO-1; 1:1000), p21 (Santa Cruz Biotechnology, sc-6246; 1:200), β-actin (Abcam, ab6276; 1:2500). Detection was performed using ImageLab (v6.0.1) with secondary goat anti-mouse IgG Fc HRP antibody (Invitrogen, A16084; 1:2500) and WesternBright Sirius chemiluminescent HRP conjugate (advansta, K-12043). β-actin was detected using goat anti-mouse Alexa-488 conjugate (Invitrogen, A-11029; 1:2500).

Supplementary Method 6: Stability measurements of recombinant p53 variants

Cancer mutations were introduced into a stabilized pseudo-wild-type variant of the human p53 DBD (residues 94-312; M133L/V203A/N239Y/N268D) that we have routinely used as a framework for biophysical and structural studies in the past[32,61]. Sequences of the pET24a-based expression vectors used are given in Supplementary Table 8. The inserts between the NdeI and EcoRI restriction sites encode for a fusion protein containing an N-terminal hexahistidine tag, the lipoyl-binding domain of the dihydrolipoamide acetyltransferase component of the pyruvate dehydrogenase complex from *Bacillus stearothermophilus (Uniprot entry P11961, residues 2-85),* followed by a TEV protease cleavage site and human p53 residues 94-312 with mutations of interest. The different p53 DBD variants were expressed

in *E. coli* C41 cells and purified by Ni-NTA column, overnight TEV protease cleavage, followed by affinity chromatography on a heparin column and size-exclusion chromatography[32].

Melting temperatures, T_m values, of the purified p53 variants were determined by differential scanning fluorimetry using an Agilent MX3005P real-time qPCR instrument (excitation/emission filters = 492/610 nm). Assay buffer: 25 mM HEPES, pH 7.5, 500 mM NaCl, 0.5 mM TCEP, with a final protein concentration of 5 µM and the fluorescent dye SYPRO Orange (Invitrogen, 10338542) at a dilution of 1:1000. The fluorescence signal was monitored upon temperature increase from 25 to 95 °C, at a heating rate of 3 °C/min, and T_m values were calculated after fitting the fluorescence curves to the Boltzmann function. Measurements were performed in three independent repeats (each consisting of four technical repeats on the same plate). Mutation-induced changes in DBD stability are given as $\Delta T_m = T_m$ (mutant) – T_m (wild type) (Supplementary Table 8). For measuring the effects of ATO on protein stability, the DBDs were incubated for 16 h at either 4 or 20 °C in assay buffer with different concentrations of ATO prior to addition of SYPRO Orange.

Supplementary Method 7: Proliferation/IC_{50} assay

4-8 x 10^4 cells were seeded on 96 well plates (Sarstedt, 83.3925) and treated with various doses of N3a after 24 h. Cells were imaged every 4 h using the IncuCyte S3 live-cell analysis system (Sartorius). Confluence data were collected using IncuCyte S3 software (v2018A) and analyzed using the area under the curve (AUC) to calculate IC_{50} values.

Supplementary Method 8: Apoptosis assay

Cells and media supernatants were collected, pelleted, and resuspended in Annexin V-APC conjugate (MabTag, AnxA100) diluted in annexin V binding buffer (BD Biosciences, 556454) according to the manufacturer's protocol. The suspension was incubated in the dark for 20 min at RT, washed in annexin V binding buffer and analyzed by flow cytometry (BD LSR II Flow Cytometer using BD FACSDiva (v6.1.3) or BD Accuri C6 Plus Cytometer using BD Accuri C6 Plus software (v1.0.23.1)). Flow cytometry data was analyzed using FlowJo (v10.8.1). For sequencing of apoptotic cells, GFP-negative cells were gated to selectively analyze cells expressing the p53 variant, i. e. cells with successful deletion of the GFP-expressing LSL cassette after AV-Cre infection. From this gate, annexin-V positive or negative cells were sorted with a Beckman Coulter MoFlo Astrios sorter using Summit (v6.3.1). See also Supplementary Figure 10.

Supplementary Method 9: Invasion/migration assays

1 x 10^5 cells were seeded in 24-well transwell inserts with 8 µm pore size (Sarstedt, 102511-1) in media containing 1% FBS[62]. The bottom well was filled with medium containing 10% FBS. After 4 d, the cells remaining on the top of the transwell membrane were thoroughly washed using a Q-Tip dipped in DPBS. The transwell inserts were then fixed for 10 min in 70% EtOH at RT, dried, and stained for 15 min with 0.2% crystal violet (Sigma-Aldrich, HT90132) in 10% EtOH. The transwell inserts were washed in water and allowed to dry. Transwell inserts were imaged using Epson Scan (v3.24G) and Adobe Photoshop CS6

(v13.0.1). For quantification, 200 µl of 20% acetic acid was added to each well and the dish was shaken for 15 min at 350 rpm at RT. The solution was then collected into 96-well plates and the absorbance was measured at 590 nm in a CYTATION 3 imaging plate reader using Gen5 software (v3.08). For normalization, 24-wells were seeded with the same number of cells in media with 1% FBS and stained one day after plating.

For invasion assays, the transwell inserts were pre-coated with 50 µl Matrigel (Corning, 354234) prior to seeding the cells. Before staining, the Matrigel was removed using a Q-Tip, and the upper part of membrane was thoroughly washed with DPBS.

In the case of siRNA transfections, the cells transfected with the ON-TARGETplus siRNA set of 4 (Horizon Discovery Ltd., LQ-003329-00 for *TP53* or D-001810-10 as non-targeting control) using Lipofectamine RNAiMax (Thermo Fisher Scientific, 3778075) according to the manufacturer's protocol and plated on the transwell inserts 24 h after siRNA transfection.

Supplementary Method 10: Animal experiments

Mouse experiments were performed in accordance with the German Animal Welfare Law (TierSchG) and received approval from the local authority (Regierungspräsidium Gießen). The mice were housed in specific-pathogen free conditions at a room temperature of 22 ± 1 °C, a relative humidity of 50 ± 10 %, a 12-h light/dark cycle and fed a standard housing diet (Altromin, 1328), with access to water *ad libitum*. The maximum allowed tumor size of 1.5 cm in diameter was not exceeded.

For *in vivo* passaging of HCT116 R175H/Δ cells, 1 x 10^6 cells were injected intravenously into the tail vein of immunodeficient $Rag2^{tm1.1Flv}$;$Il2rg^{tm1.1Flv}$ male and female mice that were a minimum of eight weeks old. Starting from the onset of clinical symptoms, mice were monitored daily using a scoring system, and were euthanized by cervical dislocation when critical symptoms appeared or after 12 weeks, whichever came first. Lungs and, if present, metastases were isolated, minced, and incubated in 1 mg/ml Collagenase/Dispase (Roche, 10269638001) and 100 µg/ml DNase I (Roche, 4536282001) at 37 °C for 1 h at 100 rpm on a horizontal shaker. The cells were filtered through a 70 µM EASYstrainer (Greiner Bio-One, 542070), pelleted at 300 x g, and erythrocytes were lysed using red blood cell lysis buffer (150 mM NH_4Cl, 10 mM $NaHCO_3$, 1.27 mM EDTA) for 5 minutes at RT. Cells were washed once with DPBS (Gibco, 14190) and cultured under standard conditions.

For the *in vivo* tumor growth and metastasis assay, HCT116 R175H/Δ p3-met cells were labelled with the intracellular Firefly (FLuc) and secreted Gaussia (GLuc) luciferases using the retroviral plasmid pMSCV_FLuc_T2A_GLuc_Hygro. For plasmid generation, the FLuc ORF was PCR-amplified using the following primers: FLuc_BglII_fw, FLuc_T2A_rev; the GLuc ORF was PCR-amplified using the following primers: GLuc_T2a_fw, GLuc_XhoI_rev. Both fragments were fused using overlap extension PCR and cloned into pCR Blunt II-TOPO vector using Zero Blunt TOPO kit (Invitrogen, 450245) according to the manufacturer's protocol. Lastly, FLuc_T2A_GLuc was cloned into MCS of pMSCV_hygro (Clontech Laboratories, Inc., 631461) using BglII (New England Biolabs, R0144) and XhoI (New England Biolabs, R0146). AmphoPack-293 cells (Takara Bio Inc., CVCL_WI47) were transfected with pMSCV-FLuc-T2A-GLuc-Hygro using calcium phosphate protocol[63]. Three days after transfection, supernatants were collected, filtered using Filtropur S 0.45 (Sarstedt, 83.1826)

and supplemented with 8 µg/ml polybrene (Sigma-Aldrich, 83.1826). 2.5 x 10^6 tumor cells were transduced with retroviral supernatant using spinoculation (1 h, 600 x g, 37°C). To knock-out p53R175H, labelled cells were transfected with pX459_blast with sgTP53_Ex3: 5'- ACT TCC TGA AAA CAA CGT TC-3' or sgTP53_Ex5: 5'-GTT GAT TCC ACA CCC CCG CC-3' and selected with 20 µg/ml blasticidin. Growth of primary, subcutaneous tumors was assessed longitudinally by measuring GLuc activity in blood samples[64]. Livers were incubated using Luciferase Cell Culture Lysis 5 x Reagent (Promega, E1531) according to the manufacturer's protocol. Firefly luciferase activity was measured in liver lysates on a plate reader luminometer (ORION II, Titertek-Berthold) using Simplicity software (v4.2) and the Beetle-Juice Luciferase assay Firefly (PJK GmbH, 102511-1) according to the manufacturer's protocol.

Supplementary Figures

Supplementary Figure 1: p53 target gene regulation in edited HCT116 cells

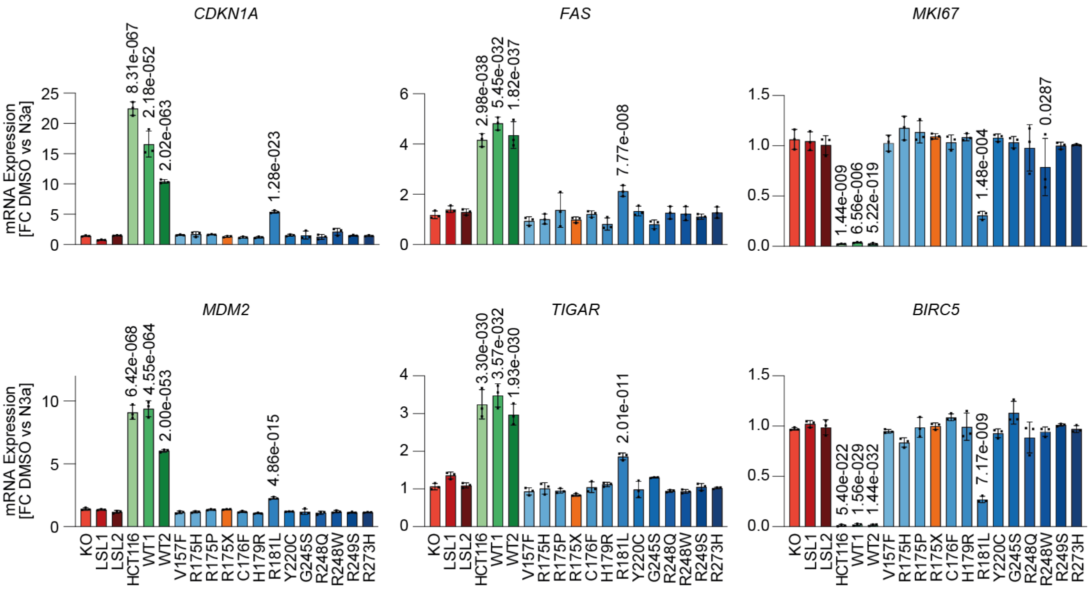

mRNA-expression changes of representative p53-activated and repressed genes following N3a-treatment in the indicated HCT116 mut/Δ cell clones. Shown is the mean ±SD. Data points indicate data from replicate RNA-seq datasets (n=3); two-way ANOVA with Sidak's post-hoc multiple comparisons test.

Supplementary Figure 2: R175 variants and Mdm2/Mdmx inhibitor responses

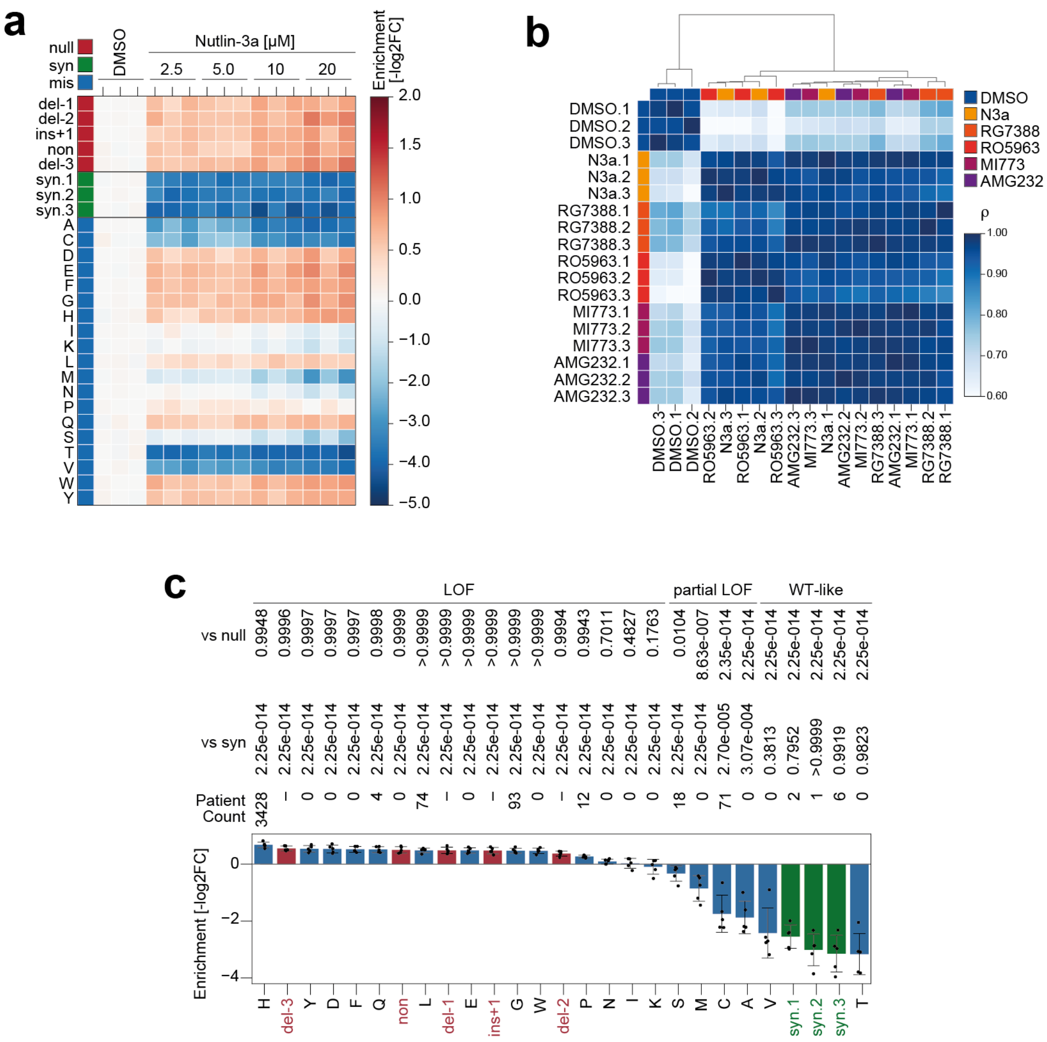

a, Dose-dependent changes in R175 variant abundance following 8 days of N3a treatment. Heatmap shows enrichment (or depletion) as the -log2 fold change versus the mean of the DMSO-treated control. n=3 biological replicates per condition. **b**, Heatmap showing pair-wise correlation coefficients (ρ, Spearman) with hierarchical clustering of samples using average linkage and Euclidean distance. **c**, Bar plot of variant enrichment/depletion shown as mean±SD for the five tested Mdm2/Mdmx inhibitors. Each data point represents the enrichment for one compound (median of 3 replicates). Null mutations are highlighted in red, synonymous variants in green. Statistical significance was tested by one-way ANOVA. Reported are p-values from Dunnett's post-hoc multiple comparisons test for each variant versus the mean of all synonymous or all null variants. For each variant, the patient sample count in the UMD mutation database is stated.

Supplementary Figure 3: Mutant p53 reactivation by APR246 and ZMC1

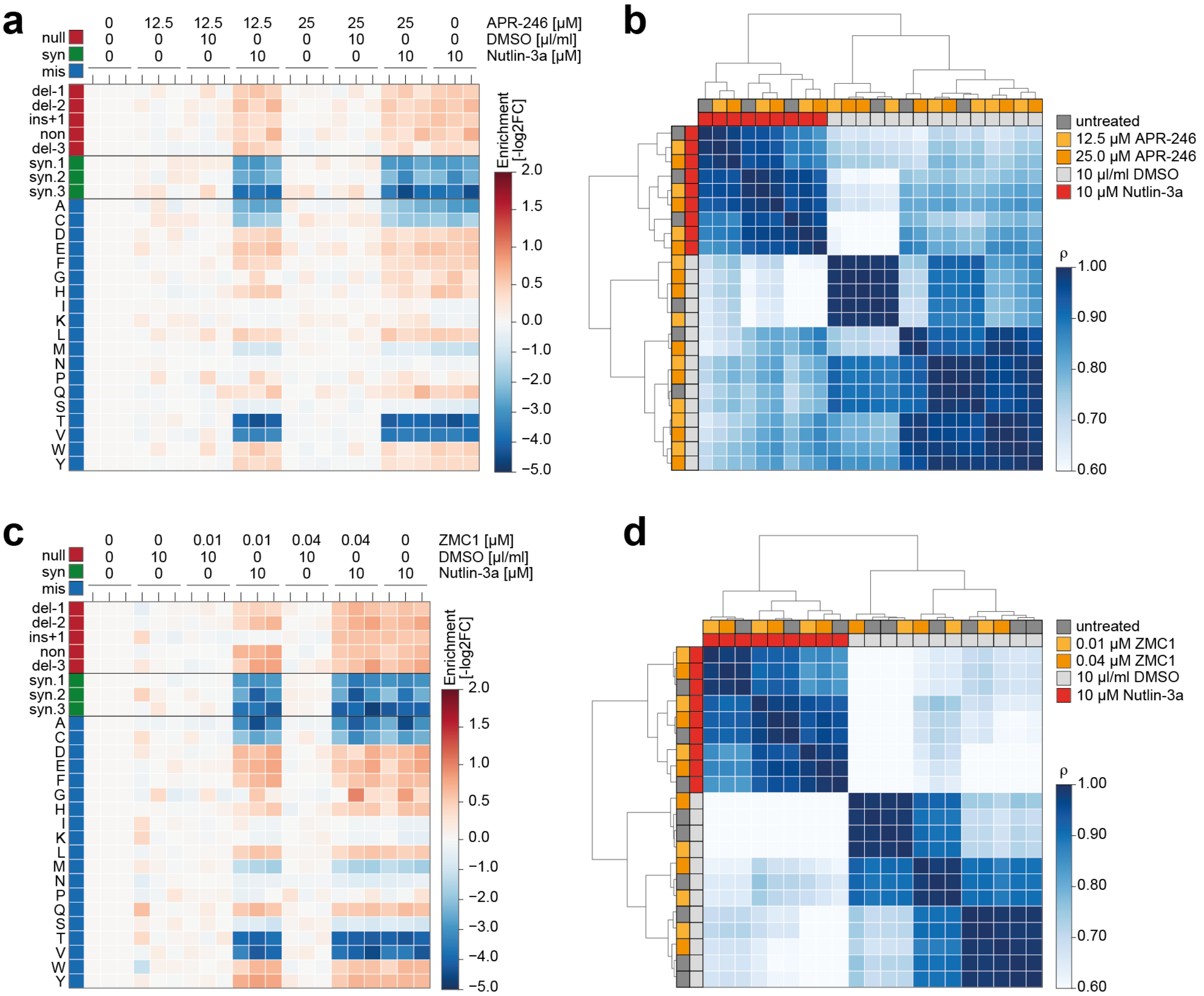

a-b, Impact of R175 variants on the cellular response to 8-day treatment with the indicated concentrations of APR-246 and N3a. **a**, Heatmap depicting changes in variant abundance as -log2 fold change versus the mean of the DMSO-treated control replicates. n=3 biological replicates per condition. **b**, Heatmap showing pair-wise correlation coefficients (ρ, Spearman). Dendrogram shows hierarchical clustering of samples using average linkage and Euclidean distance. **c-d**, Impact of R175 variants on the cellular response to 8-day treatment with the indicated concentrations of ZMC1 and N3a. **c**, Heatmap depicting changes in variant abundance as -log2 fold change versus the mean of the DMSO-treated control replicates. n=3 biological replicates per condition. **d**, Heatmap showing pair-wise correlation coefficients (ρ, Spearman). Dendrogram shows hierarchical clustering of samples using average linkage and Euclidean distance.

Supplementary Figure 4: Separation-of-function phenotype of R175 variants

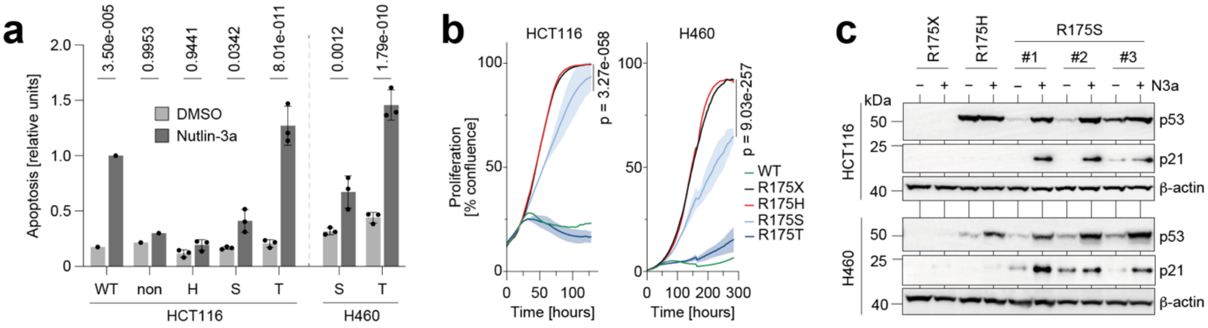

Analysis of N3a response in HCT116 mut/Δ and H460 mut/Δ/Δ clones with indicated codon R175H/S/T/X mutations. **a**, Apoptosis determined 48 h after N3a treatment by annexin V flow cytometry. Shown is the percentage of annexin V+ cells normalized to respective WT cells as mean ±SD of n=3 independent R175S and R175T clones. Statistical analysis was performed using two-way ANOVA with Sidak's post-hoc multiple comparisons test. **b**, Proliferation curves of cells with indicated genotype in the presence of N3a. Shown is the confluence as the mean percentage ±SD of n=3 independent R175S and R175T clones. Statistical analysis was performed using two-way ANOVA with Sidak's post-hoc multiple comparisons test. **c**, Western blot demonstrating mutant p53 and p21 protein expression in independent HCT116 and H460 clones after 4 days in the absence and presence of N3a. Results are representative of two independent blots.

Supplementary Figure 5: DBD variant coverage in *TP53* DMS studies

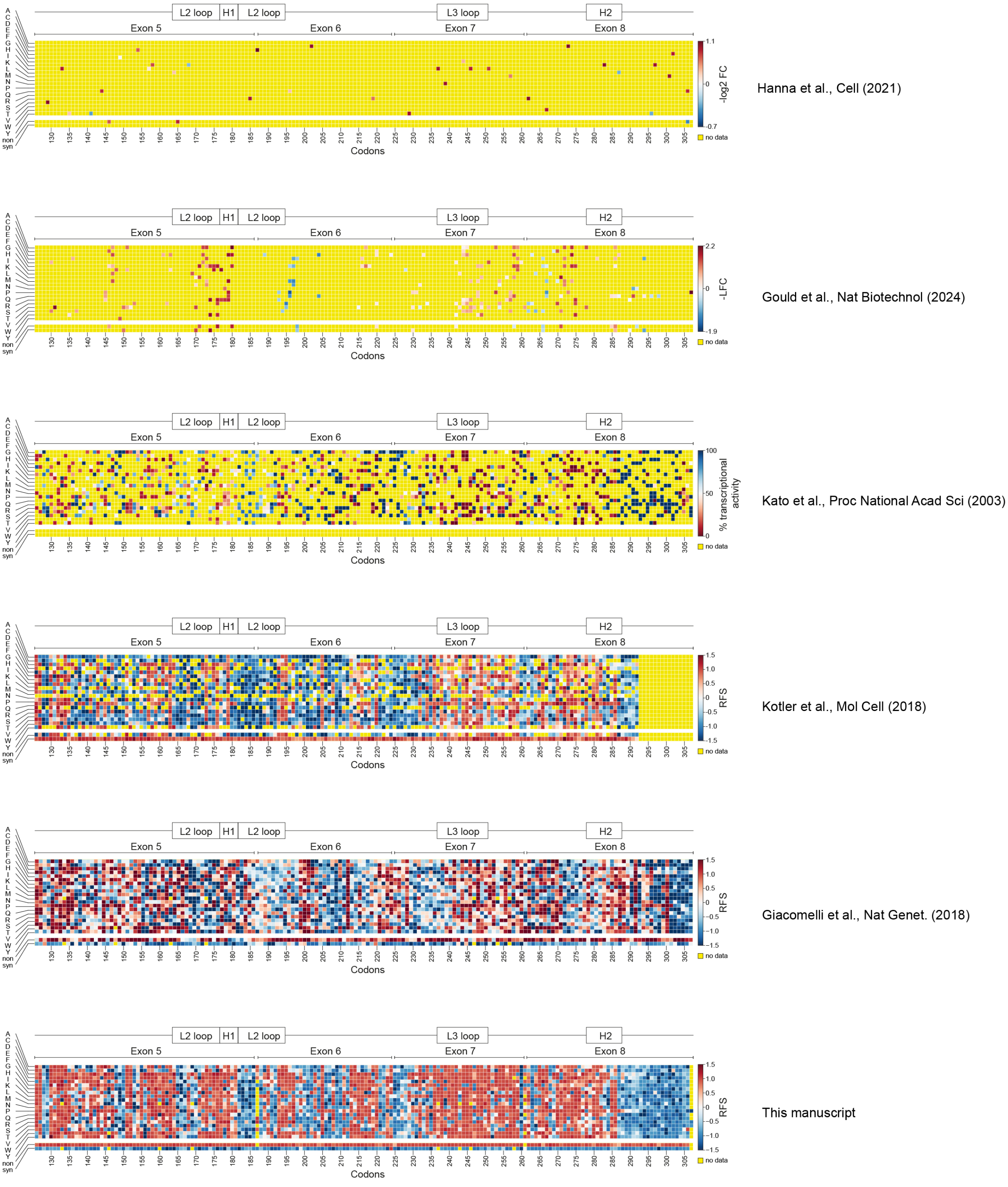

Heatmaps show the RFS for all missense, synonymous (syn), and nonsense (non) variants reported by the respective studies using cDNA overexpression (Kato et al., 2003[19], Kotler et al., 2018[20], Giacomelli et al., 2018[21]), CRISPR base editing (Hanna et al., 2021[65]), CRISPR prime editing (Gould et al., 2024[66]) and CRISPR-HDR (this manuscript). Missing values are shown in yellow.

Supplementary Figure 6: CRISPR RFS scores versus transcriptional activity

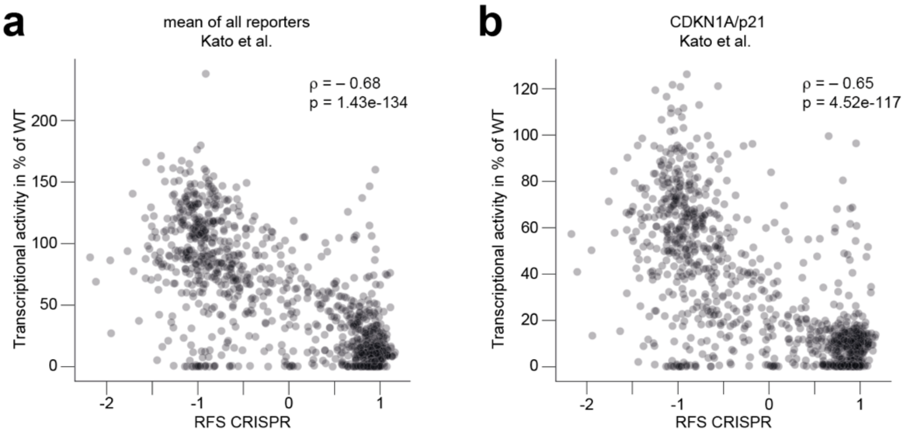

a-b, Scatter plots illustrate the negative correlation between CRISPR RFS values and transcriptional activity measured in yeast (Kato et al., 2003[19]; activity in % of WT). (**a**) Mean activity of all eight reporters, (**b**) activity of the CDKN1A/p21/WAF1 reporter. ρ, Spearman correlation coefficient with p-value approximated using a two-tailed t-distribution.

Supplementary Figure 7: CRISPR versus cDNA overexpression screen

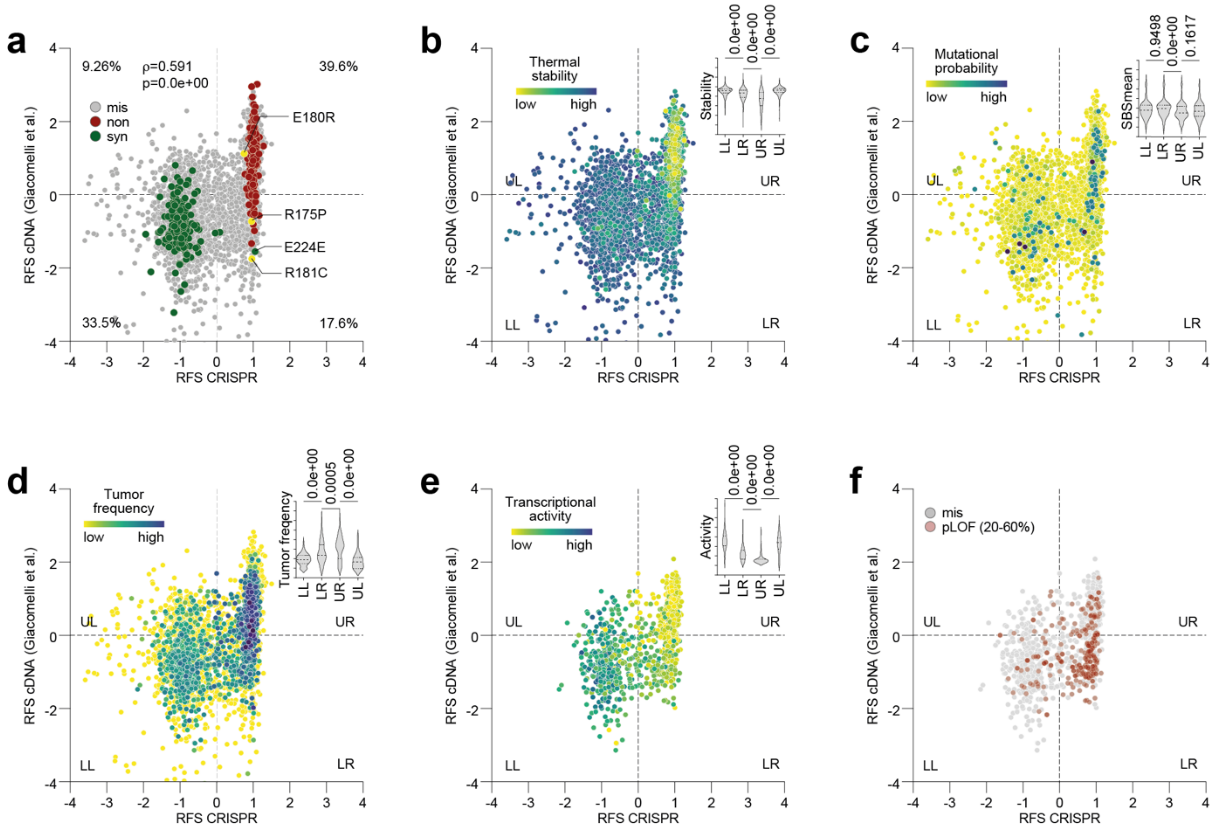

Scatter plots illustrate the correlation between RFS values obtained by CRISPR mutagenesis and cDNA overexpression (Giacomelli et al., 2018[21]). Variants are colored based on (**a**) mutation type, (**b**) thermal stability as predicted by HoTMuSiC[67] , (**c**) average mutational probability using a weighted average mutational signature SBSmean, (**d**) frequency in cancer patients (sum of all records in UMD, IARC/NCI, TCGA, GENIE databases), (**e**) mean transcriptional activity relative to wild-type p53 (WT) as measured in a yeast-based reporter system (Kato et al., 2003[19]), and (**f**) classification as pLOF (20-60% transcriptional activity). Inserted violin plots illustrate the value distribution in the four quadrants (LL, lower left; LR, lower right; UL, upper left; UR, upper right) and report p values from one-way ANOVA with Tukey's post-hoc multiple comparisons tests (***, <0.001; ns, not significant). ρ, Spearman correlation coefficient with p-value approximated using a two-tailed t-distribution.

Supplementary Figure 8: Temperature-sensitive function of LR variants

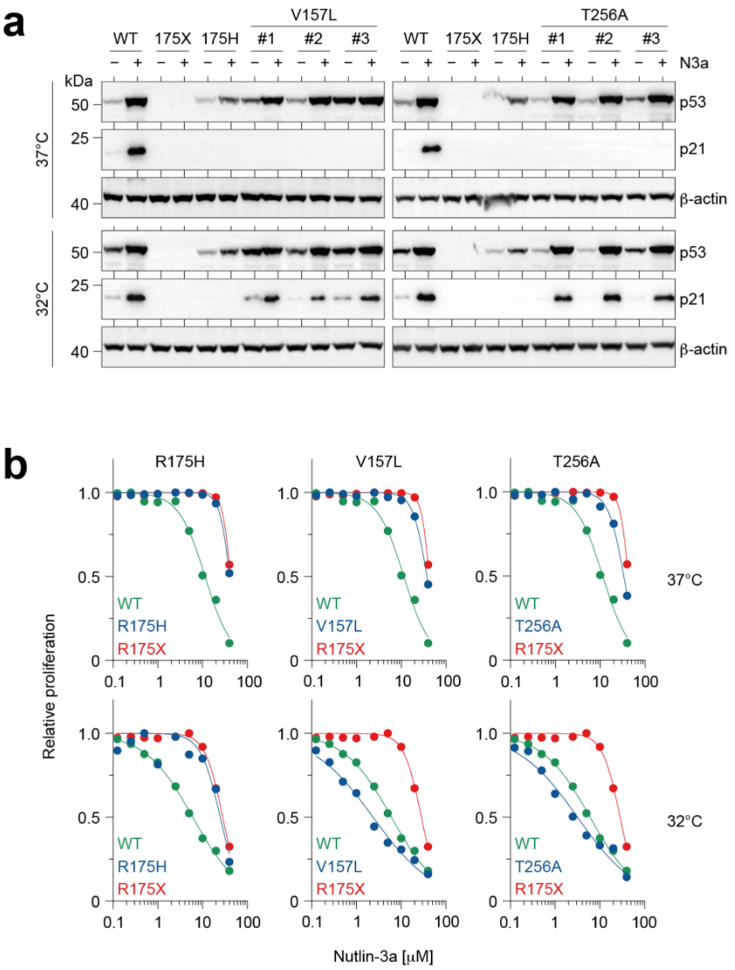

Indicated variants were introduced into HCT116 LSL/Δ cells by CRISPR-HDR. **a**, Western blot of indicated HCT116 mut/Δ cell clones cultured with or without 10 μM N3a at 37 °C or 32 °C. Results are representative of two independent blots. **b**, Dose-response curves of N3a for representative cell clones, shown as mean relative proliferation (n=4 replicates), based on confluence measurements using real-time live-cell imaging.

Supplementary Figure 9: Fitness impact of variants near exon-intron borders

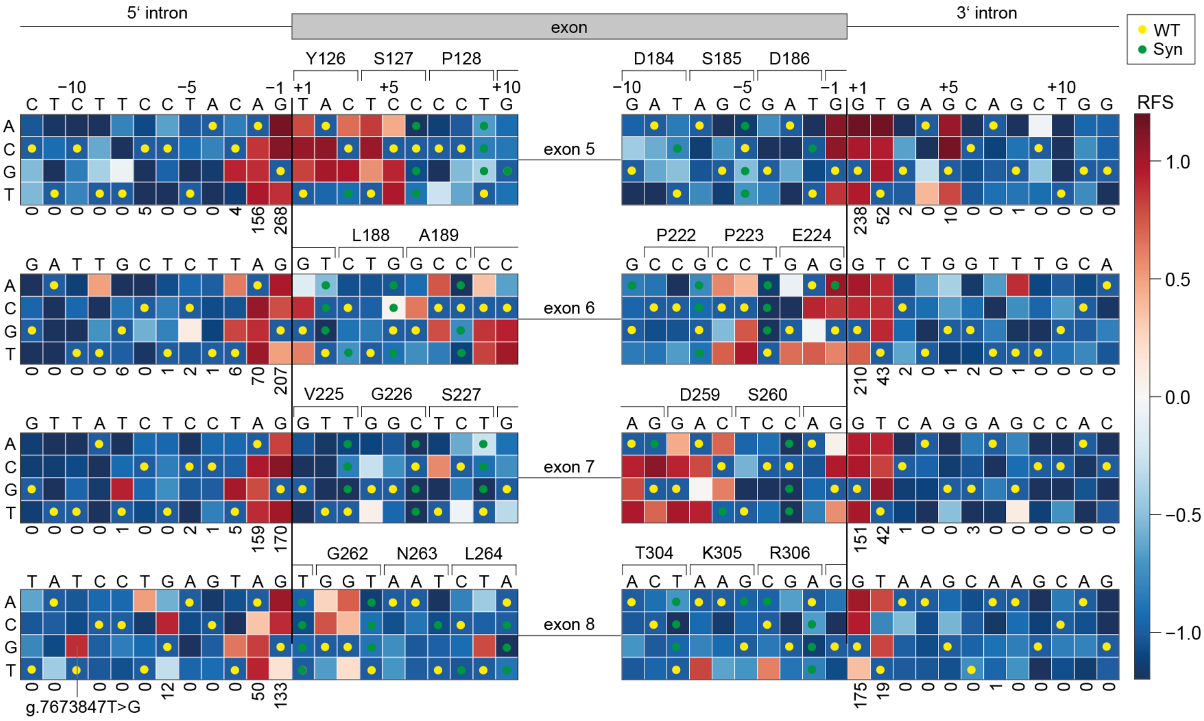

Heatmaps show the RFS of indicated single-nucleotide substitution variants. Wild-type and synonymous variants are indicated with yellow and green dots, respectively. The number of cancer patients in GENIE with mutations at exon-flanking intronic nucleotides is indicated below the heatmaps.

Supplementary Figure 10: Gating strategy for apoptosis analysis

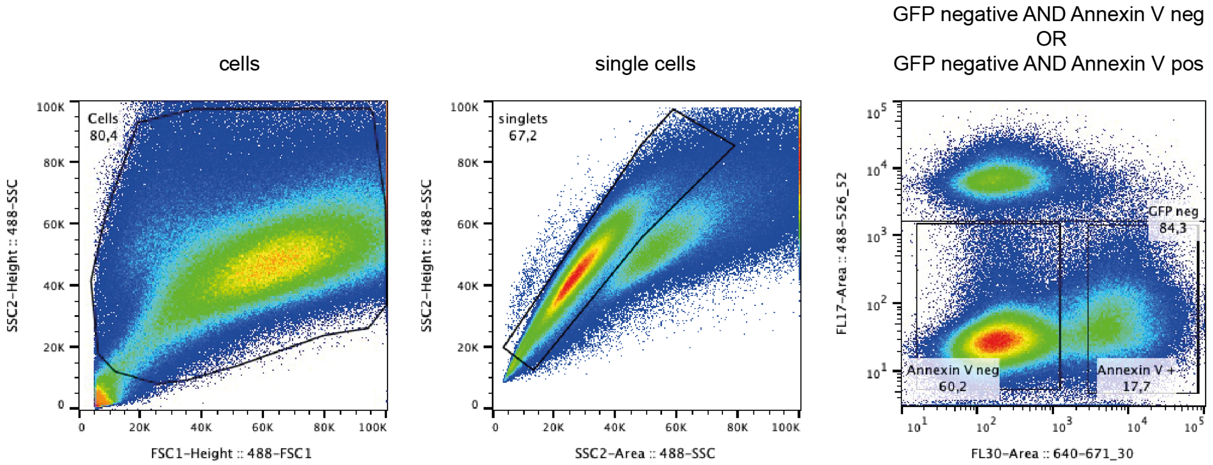

Events were gated for SSC-H and FSC-H to exclude debris and FSC-H and FSC-A to isolate single cells. Using APC-A (annexin-V) and FITC-A (GFP) channels, GFP negative (Cre-recombined) cells were sorted into an annexin-V positive and annexin-V negative population for further analysis by sequencing. Unrecombined, completely recombined and unstained cells were used as controls to set up the gating.

Supplementary Figure 11: Unprocessed Blots for Supplementary Figure 4c

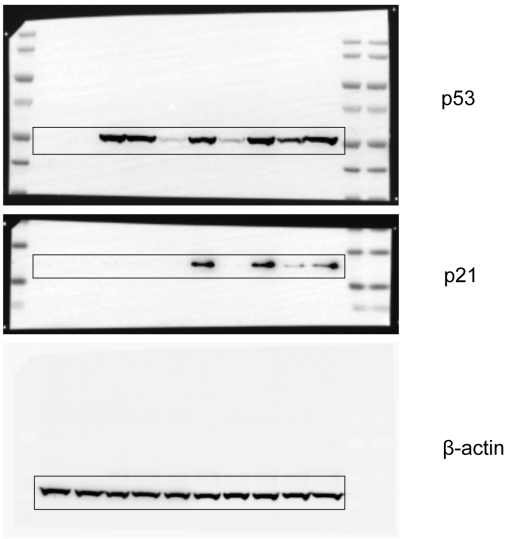

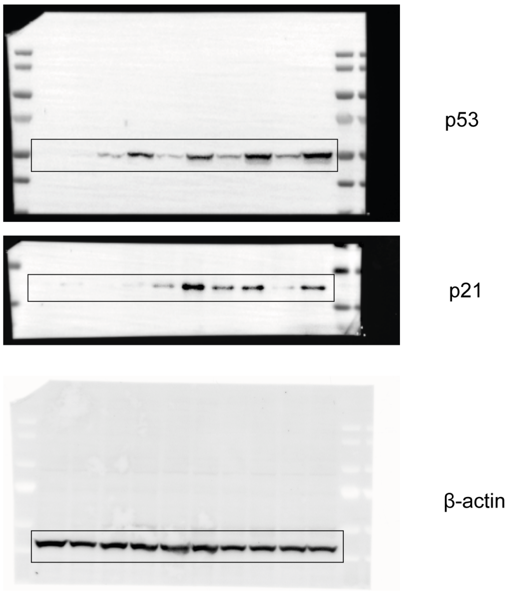

Supplementary Figure 12: Unprocessed Blots for Supplementary Figure 8a

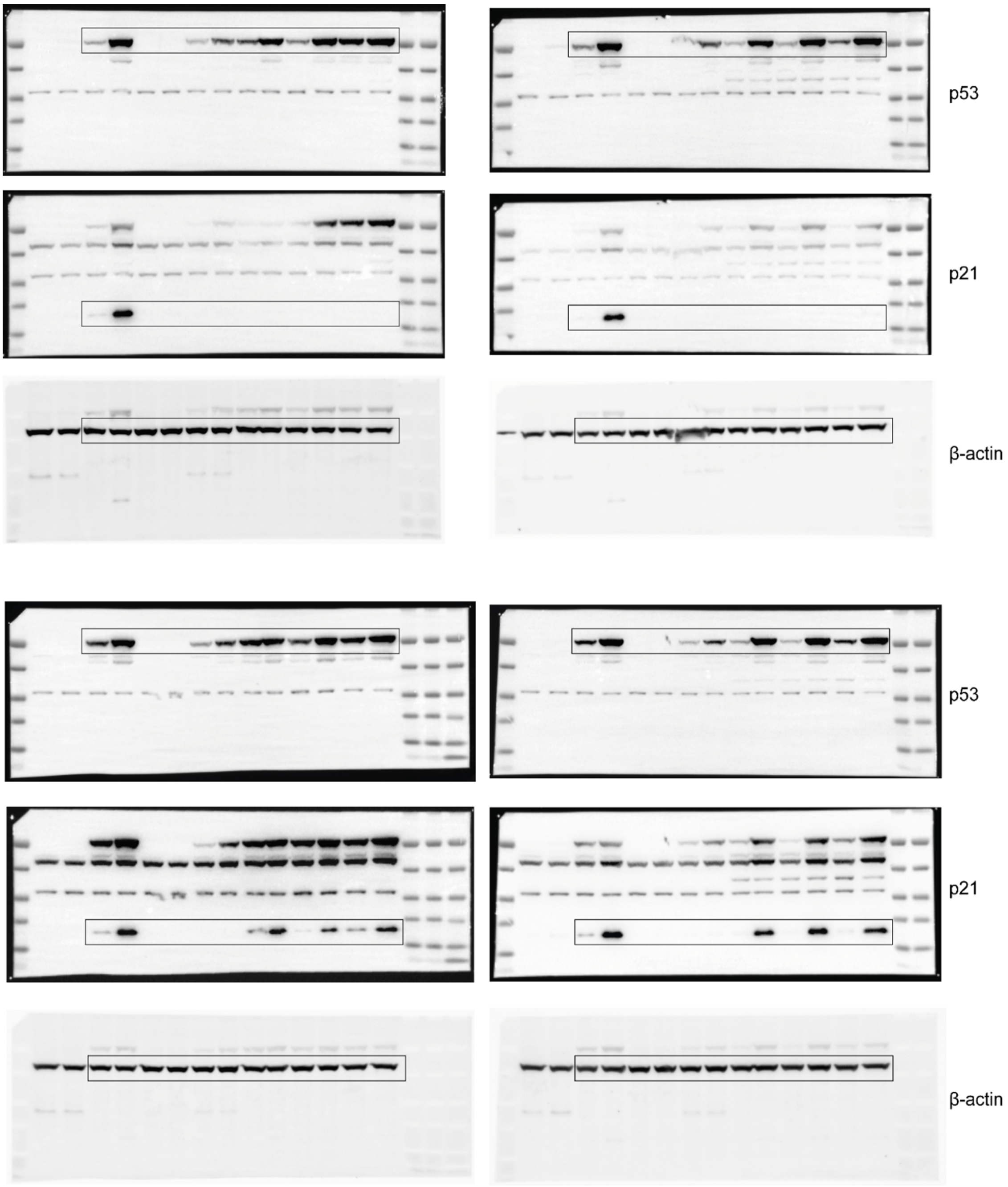

Supplementary Information References

1 Bunz, F. *et al.* Requirement for p53 and p21 to sustain G2 arrest after DNA damage. *Science* **282**, 1497-1501 (1998). https://doi.org/10.1126/science.282.5393.1497
2 Nakano, K. & Vousden, K. H. PUMA, a Novel Proapoptotic Gene, Is Induced by p53. *Mol. Cell* **7**, 683-694 (2001). https://doi.org/10.1016/s1097-2765(01)00214-3
3 Bensaad, K. *et al.* TIGAR, a p53-inducible regulator of glycolysis and apoptosis. *Cell* **126**, 107-120 (2006). https://doi.org/10.1016/j.cell.2006.05.036
4 Maddocks, O. D. *et al.* Serine starvation induces stress and p53-dependent metabolic remodelling in cancer cells. *Nature* **493**, 542-546 (2013). https://doi.org/10.1038/nature11743
5 Ihry, R. J. *et al.* p53 inhibits CRISPR-Cas9 engineering in human pluripotent stem cells. *Nat. Med.* **24**, 939-946 (2018). https://doi.org/10.1038/s41591-018-0050-6
6 Haapaniemi, E., Botla, S., Persson, J., Schmierer, B. & Taipale, J. CRISPR-Cas9 genome editing induces a p53-mediated DNA damage response. *Nat. Med.* (2018). https://doi.org/10.1038/s41591-018-0049-z
7 Enache, O. M. *et al.* Cas9 activates the p53 pathway and selects for p53-inactivating mutations. *Nat. Genet.* **52**, 662-668 (2020). https://doi.org/10.1038/s41588-020-0623-4
8 Findlay, G. M., Boyle, E. A., Hause, R. J., Klein, J. C. & Shendure, J. Saturation editing of genomic regions by multiplex homology-directed repair. *Nature* **513**, 120-123 (2014). https://doi.org/10.1038/nature13695
9 Maes, S., Deploey, N., Peelman, F. & Eyckerman, S. Deep mutational scanning of proteins in mammalian cells. *Cell Reports Methods* **3** (2023). https://doi.org/10.1016/j.crmeth.2023.100641
10 Tabet, D., Parikh, V., Mali, P., Roth, F. P. & Claussnitzer, M. Scalable Functional Assays for the Interpretation of Human Genetic Variation. *Annu. Rev. Genet.* **56**, 441-465 (2022). https://doi.org/10.1146/annurev-genet-072920-032107
11 Muller, P. A. & Vousden, K. H. Mutant p53 in cancer: new functions and therapeutic opportunities. *Cancer Cell* **25**, 304-317 (2014). https://doi.org/10.1016/j.ccr.2014.01.021
12 Terzian, T. *et al.* The inherent instability of mutant p53 is alleviated by Mdm2 or p16INK4a loss. *Genes Dev.* **22**, 1337-1344 (2008). https://doi.org/10.1101/gad.1662908
13 Redman-Rivera, L. N. *et al.* Acquisition of aneuploidy drives mutant p53-associated gain-of-function phenotypes. *Nat Commun* **12**, 5184 (2021). https://doi.org/10.1038/s41467-021-25359-z
14 Isermann, T. *et al.* Suppression of HSF1 activity by wildtype p53 creates a driving force for p53 loss-of-heterozygosity. *Nat Commun* **12**, 4019 (2021). https://doi.org/10.1038/s41467-021-24064-1
15 Lang, G. A. *et al.* Gain of function of a p53 hot spot mutation in a mouse model of Li-Fraumeni syndrome. *Cell* **119**, 861-872 (2004). https://doi.org/10.1016/j.cell.2004.11.006
16 Muller, P. A. *et al.* Mutant p53 drives invasion by promoting integrin recycling. *Cell* **139**, 1327-1341 (2009). https://doi.org/10.1016/j.cell.2009.11.026
17 Greenman, C. *et al.* Patterns of somatic mutation in human cancer genomes. *Nature* **446**, 153-158 (2007). https://doi.org/10.1038/nature05610
18 Alexandrov, L. B. *et al.* The repertoire of mutational signatures in human cancer. *Nature* **578**, 94-101 (2020). https://doi.org/10.1038/s41586-020-1943-3

19 Kato, S. *et al.* Understanding the function-structure and function-mutation relationships of p53 tumor suppressor protein by high-resolution missense mutation analysis. *Proc. Natl. Acad. Sci. U. S. A.* **100**, 8424-8429 (2003). https://doi.org/10.1073/pnas.1431692100
20 Kotler, E. *et al.* A Systematic p53 Mutation Library Links Differential Functional Impact to Cancer Mutation Pattern and Evolutionary Conservation. *Mol. Cell* **71**, 178-190 e178 (2018). https://doi.org/10.1016/j.molcel.2018.06.012
21 Giacomelli, A. O. *et al.* Mutational processes shape the landscape of TP53 mutations in human cancer. *Nat. Genet.* **50**, 1381-1387 (2018). https://doi.org/10.1038/s41588-018-0204-y
22 Cao, S. *et al.* Discovery of driver non-coding splice-site-creating mutations in cancer. *Nat Commun* **11**, 5573 (2020). https://doi.org/10.1038/s41467-020-19307-6
23 Kim, J. *et al.* A framework for individualized splice-switching oligonucleotide therapy. *Nature* **619**, 828-836 (2023). https://doi.org/10.1038/s41586-023-06277-0
24 Fayer, S. *et al.* Closing the gap: Systematic integration of multiplexed functional data resolves variants of uncertain significance in BRCA1, TP53, and PTEN. *The American Journal of Human Genetics* **108**, 2248-2258 (2021). https://doi.org/10.1016/j.ajhg.2021.11.001
25 Brnich, S. E. *et al.* Recommendations for application of the functional evidence PS3/BS3 criterion using the ACMG/AMP sequence variant interpretation framework. *Genome Med.* **12** (2019). https://doi.org/10.1186/s13073-019-0690-2
26 Gelman, H. *et al.* Recommendations for the collection and use of multiplexed functional data for clinical variant interpretation. *Genome Med.* **11** (2019). https://doi.org/10.1186/s13073-019-0698-7
27 Richards, S. *et al.* Standards and guidelines for the interpretation of sequence variants: a joint consensus recommendation of the American College of Medical Genetics and Genomics and the Association for Molecular Pathology. *Genet. Med.* **17**, 405-424 (2015). https://doi.org/10.1038/gim.2015.30
28 Bushman, F. *et al.* Genome-wide analysis of retroviral DNA integration. *Nat. Rev. Microbiol.* **3**, 848-858 (2005). https://doi.org/10.1038/nrmicro1263
29 Liu, G. *et al.* Chromosome stability, in the absence of apoptosis, is critical for suppression of tumorigenesis in Trp53 mutant mice. *Nat. Genet.* **36**, 63-68 (2004). https://doi.org/10.1038/ng1282
30 Timofeev, O. *et al.* p53 DNA binding cooperativity is essential for apoptosis and tumor suppression in vivo. *Cell Rep.* **3**, 1512-1525 (2013). https://doi.org/10.1016/j.celrep.2013.04.008
31 Kang, J. G. *et al.* A Mouse Homolog of a Human TP53 Germline Mutation Reveals a Lipolytic Activity of p53. *Cell Rep.* **30**, 783-792 e785 (2020). https://doi.org/10.1016/j.celrep.2019.12.074
32 Bauer, M. R. *et al.* Targeting Cavity-Creating p53 Cancer Mutations with Small-Molecule Stabilizers: the Y220X Paradigm. *ACS Chem. Biol.* **15**, 657-668 (2020). https://doi.org/10.1021/acschembio.9b00748
33 Balourdas, D. I., Markl, A. M., Kramer, A., Settanni, G. & Joerger, A. C. Structural basis of p53 inactivation by cavity-creating cancer mutations and its implications for the development of mutant p53 reactivators. *Cell Death Dis.* **15**, 408 (2024). https://doi.org/10.1038/s41419-024-06739-x
34 Bullock, A. N. & Fersht, A. R. Rescuing the function of mutant p53. *Nat. Rev. Cancer* **1**, 68-76 (2001). https://doi.org/10.1038/35094077

35 Joerger, A. C. & Fersht, A. R. The p53 Pathway: Origins, Inactivation in Cancer, and Emerging Therapeutic Approaches. *Annu. Rev. Biochem.* **85**, 375-404 (2016). https://doi.org/10.1146/annurev-biochem-060815-014710
36 Tang, Y. *et al.* Repurposing antiparasitic antimonials to noncovalently rescue temperature-sensitive p53 mutations. *Cell Rep.* **39**, 110622 (2022). https://doi.org/10.1016/j.celrep.2022.110622
37 Lu, J., Chen, L., Song, Z., Das, M. & Chen, J. Hypothermia Effectively Treats Tumors with Temperature-Sensitive p53 Mutations. *Cancer Res.* **81**, 3905-3915 (2021). https://doi.org/10.1158/0008-5472.CAN-21-0033
38 Supek, F., Minana, B., Valcarcel, J., Gabaldon, T. & Lehner, B. Synonymous mutations frequently act as driver mutations in human cancers. *Cell* **156**, 1324-1335 (2014). https://doi.org/10.1016/j.cell.2014.01.051
39 Carbonnier, V., Leroy, B., Rosenberg, S. & Soussi, T. Comprehensive assessment of TP53 loss of function using multiple combinatorial mutagenesis libraries. *Sci. Rep.* **10**, 20368 (2020). https://doi.org/10.1038/s41598-020-74892-2
40 Smeby, J. *et al.* Transcriptional and functional consequences of TP53 splice mutations in colorectal cancer. *Oncogenesis* **8**, 35 (2019). https://doi.org/10.1038/s41389-019-0141-3
41 Chui, M. H. *et al.* Somatic intronic TP53 c.375+5G mutations are a recurrent but under-recognized mode of TP53 inactivation. *J Pathol Clin Res* **8**, 14-18 (2022). https://doi.org/10.1002/cjp2.242
42 Bradley, R. K. & Anczukow, O. RNA splicing dysregulation and the hallmarks of cancer. *Nat. Rev. Cancer* (2023). https://doi.org/10.1038/s41568-022-00541-7
43 Freed-Pastor, W. A. & Prives, C. Mutant p53: one name, many proteins. *Genes Dev.* **26**, 1268-1286 (2012). https://doi.org/10.1101/gad.190678.112
44 Donehower, L. A. *et al.* Integrated Analysis of TP53 Gene and Pathway Alterations in The Cancer Genome Atlas. *Cell Rep.* **28**, 1370-1384 e1375 (2019). https://doi.org/10.1016/j.celrep.2019.07.001
45 Romanovsky, E. *et al.* Homogenous TP53mut-associated tumor biology across mutation and cancer types revealed by transcriptome analysis. *Cell Death Discov* **9**, 126 (2023). https://doi.org/10.1038/s41420-023-01413-1
46 Alexandrova, E. M. *et al.* Improving survival by exploiting tumour dependence on stabilized mutant p53 for treatment. *Nature* **523**, 352-356 (2015). https://doi.org/10.1038/nature14430
47 Schulz-Heddergott, R. *et al.* Therapeutic Ablation of Gain-of-Function Mutant p53 in Colorectal Cancer Inhibits Stat3-Mediated Tumor Growth and Invasion. *Cancer Cell* **34**, 298-314 e297 (2018). https://doi.org/10.1016/j.ccell.2018.07.004
48 Lu, Y. *et al.* Genome-wide CRISPR screens identify novel regulators of wild-type and mutant p53 stability. *Mol. Syst. Biol.* **20**, 719-740 (2024). https://doi.org/10.1038/s44320-024-00032-x
49 Milner, J., Medcalf, E. A. & Cook, A. C. Tumor suppressor p53: analysis of wild-type and mutant p53 complexes. *Mol. Cell. Biol.* **11**, 12-19 (1991).
50 Milner, J. & Medcalf, E. A. Cotranslation of activated mutant p53 with wild type drives the wild-type p53 protein into the mutant conformation. *Cell* **65**, 765-774 (1991).
51 Shetzer, Y. *et al.* The onset of p53 loss of heterozygosity is differentially induced in various stem cell types and may involve the loss of either allele. *Cell Death Differ.* **21**, 1419-1431 (2014). https://doi.org/10.1038/cdd.2014.57
52 Lowe, S. W., Ruley, H. E., Jacks, T. & Housman, D. E. p53-dependent apoptosis modulates the cytotoxicity of anticancer agents. *Cell* **74**, 957-967 (1993).

53 Bunz, F. *et al.* Disruption of p53 in human cancer cells alters the responses to therapeutic agents. *J. Clin. Invest.* **104**, 263-269 (1999). https://doi.org/10.1172/JCI6863
54 Rago, C., Vogelstein, B. & Bunz, F. Genetic knockouts and knockins in human somatic cells. *Nat. Protoc.* **2**, 2734-2746 (2007). https://doi.org/10.1038/nprot.2007.408
55 Chen, P. J. *et al.* Enhanced prime editing systems by manipulating cellular determinants of editing outcomes. *Cell* **184**, 5635-5652 e5629 (2021). https://doi.org/10.1016/j.cell.2021.09.018
56 Dobin, A. *et al.* STAR: ultrafast universal RNA-seq aligner. *Bioinformatics* **29**, 15-21 (2013). https://doi.org/10.1093/bioinformatics/bts635
57 Smith, T., Heger, A. & Sudbery, I. UMI-tools: modeling sequencing errors in Unique Molecular Identifiers to improve quantification accuracy. *Genome Res.* **27**, 491-499 (2017). https://doi.org/10.1101/gr.209601.116
58 Love, M. I., Huber, W. & Anders, S. Moderated estimation of fold change and dispersion for RNA-seq data with DESeq2. *Genome Biol.* **15**, 550 (2014). https://doi.org/10.1186/s13059-014-0550-8
59 Subramanian, A. *et al.* Gene set enrichment analysis: a knowledge-based approach for interpreting genome-wide expression profiles. *Proc. Natl. Acad. Sci. U. S. A.* **102**, 15545-15550 (2005). https://doi.org/10.1073/pnas.0506580102
60 Fennell, K. A. *et al.* Non-genetic determinants of malignant clonal fitness at single-cell resolution. *Nature* **601**, 125-131 (2022). https://doi.org/10.1038/s41586-021-04206-7
61 Joerger, A. C., Ang, H. C. & Fersht, A. R. Structural basis for understanding oncogenic p53 mutations and designing rescue drugs. *Proc. Natl. Acad. Sci. U. S. A.* **103**, 15056-15061 (2006). https://doi.org/10.1073/pnas.0607286103
62 Pavlakis, E. *et al.* Mutant p53-ENTPD5 control of the calnexin/calreticulin cycle: a druggable target for inhibiting integrin-alpha5-driven metastasis. *J. Exp. Clin. Cancer Res.* **42**, 203 (2023). https://doi.org/10.1186/s13046-023-02785-z
63 Kutner, R. H., Zhang, X. Y. & Reiser, J. Production, concentration and titration of pseudotyped HIV-1-based lentiviral vectors. *Nat. Protoc.* **4**, 495-505 (2009). https://doi.org/10.1038/nprot.2009.22
64 Merle, N. *et al.* Monitoring autochthonous lung tumors induced by somatic CRISPR gene editing in mice using a secreted luciferase. *Mol. Cancer* **21**, 191 (2022). https://doi.org/10.1186/s12943-022-01661-2
65 Hanna, R. E. *et al.* Massively parallel assessment of human variants with base editor screens. *Cell* **184**, 1064-1080 e1020 (2021). https://doi.org/10.1016/j.cell.2021.01.012
66 Gould, S. I. *et al.* High-throughput evaluation of genetic variants with prime editing sensor libraries. *Nat. Biotechnol.* (2024). https://doi.org/10.1038/s41587-024-02172-9
67 Pucci, F., Bourgeas, R. & Rooman, M. Predicting protein thermal stability changes upon point mutations using statistical potentials: Introducing HoTMuSiC. *Sci. Rep.* **6**, 23257 (2016). https://doi.org/10.1038/srep23257
