# Supplementary material for: Deep CRISPR mutagenesis characterizes the functional diversity of TP53 mutations
Source: Nat Genet. 2025 Jan 7;57(1):140–53. doi: 10.1038/s41588-024-02039-4 (PMC11735402; doi:10.1038/s41588-024-02039-4)
Supplement: Supplementary file 2 — Reporting Summary [file 41588_2024_2039_MOESM2_ESM.pdf]

nature portfolio

Corresponding author(s): Thorsten Stiewe

Last updated by author(s): Oct 23, 2024

# Reporting Summary

Nature Portfolio wishes to improve the reproducibility of the work that we publish. This form provides structure for consistency and transparency in reporting. For further information on Nature Portfolio policies, see our Editorial Policies and the Editorial Policy Checklist.

## Statistics

For all statistical analyses, confirm that the following items are present in the figure legend, table legend, main text, or Methods section.

| n/a | Confirmed | |
|---|---|---|
| ☐ | ☒ | The exact sample size (*n*) for each experimental group/condition, given as a discrete number and unit of measurement |
| ☐ | ☒ | A statement on whether measurements were taken from distinct samples or whether the same sample was measured repeatedly |
| ☐ | ☒ | The statistical test(s) used AND whether they are one- or two-sided<br>*Only common tests should be described solely by name; describe more complex techniques in the Methods section.* |
| ☒ | ☐ | A description of all covariates tested |
| ☐ | ☒ | A description of any assumptions or corrections, such as tests of normality and adjustment for multiple comparisons |
| ☐ | ☒ | A full description of the statistical parameters including central tendency (e.g. means) or other basic estimates (e.g. regression coefficient) AND variation (e.g. standard deviation) or associated estimates of uncertainty (e.g. confidence intervals) |
| ☐ | ☒ | For null hypothesis testing, the test statistic (e.g. *F*, *t*, *r*) with confidence intervals, effect sizes, degrees of freedom and *P* value noted<br>*Give P values as exact values whenever suitable.* |
| ☒ | ☐ | For Bayesian analysis, information on the choice of priors and Markov chain Monte Carlo settings |
| ☒ | ☐ | For hierarchical and complex designs, identification of the appropriate level for tests and full reporting of outcomes |
| ☐ | ☒ | Estimates of effect sizes (e.g. Cohen's *d*, Pearson's *r*), indicating how they were calculated |

*Our web collection on statistics for biologists contains articles on many of the points above.*

## Software and code

Policy information about availability of computer code

| | |
|---|---|
| Data collection | ImageLab (v6.0.1)<br>IncuCyte S3 Software (v2018A)<br>BD FACSDiva (v6.1.3)<br>BD Accuri C6 Plus (v1.0.23.1)<br>Summit (v6.3.1)<br>Simplicity (v4.2)<br>Epson Scan (v3.24G)<br>Gen5 (v3.08) |
| Data analysis | GraphPad Prism (9.4.1)<br>Adobe Photoshop CS6 (v13.0.1)<br>Microsoft Excel 2019 (v2301)<br>LightCycler 480 Software (v1.5.0.39)<br>mmdemultiplex (v0.1): https://github.com/MarcoMernberger/mmdemultiplex.git<br>CutAdapt (v3.5)<br>NGmerge (v0.3)<br>STAR (v2.7.10a)<br>UMI-tools (v1.1.1)<br>DEseq2 (v1.34.0)<br>Enrich2 (v1.2.0)<br>GSEA (v4.2.2) |

ScanPy (v1.9.0)
IncuCyte S3 Software (v2018A)
FlowJo (v10.8.1)
ConSurf: https://consurf.tau.ac.il/consurf_index.php (Ben Chorin et al., 2020; doi: 10.1002/pro.3779)
PyMOL (v2.5.2)
ProteinTools: https://proteintools.uni-bayreuth.de (Ferruz et al., 2021; doi: 10.1093/nar/gkab375)
HoTMuSiC: https://soft.dezyme.com (Pucci et al., 2016; doi: 10.1038/srep23257)
Python (v3.9.12) with Matplotlib (v3.5.1), Seaborn (v0.11.2), SciPy (v1.7.3), Statsmodels (v0.13.2)
Adobe Illustrator (26.5.2)

Analysis code is available on GitHub: https://github.com/IMTMarburg/TP53_SGE

For manuscripts utilizing custom algorithms or software that are central to the research but not yet described in published literature, software must be made available to editors and reviewers. We strongly encourage code deposition in a community repository (e.g. GitHub). See the Nature Portfolio guidelines for submitting code & software for further information.

## Data

Policy information about availability of data

All manuscripts must include a data availability statement. This statement should provide the following information, where applicable:

- Accession codes, unique identifiers, or web links for publicly available datasets
- A description of any restrictions on data availability
- For clinical datasets or third party data, please ensure that the statement adheres to our policy

Raw data generated or analyzed for the present study are available as Source Data files. Variant library designs are available as Supplementary Tables S1-S10. For comparison with cDNA-based mutome screens data was used from Kotler et al. 2018 (Supplemental Table 2, RFS_H1299) and from Giacomelli et al., 2018 (Supplementary Table 3, A549_p53NULL_Nutlin-3_Z-score). Publicly available datasets used during this study were UMD TP53 Mutation Database (2017_r2), NCI/IARC The TP53 Database (R20, July 2019), curated set of non-redundant studies (TCGA) and the AACR project GENIE (downloaded from cBioPortal on Dec 20, 2022), Mutational Signatures (v3.3, June 2022, downloaded from COSMIC), ClinVar (downloaded from https://www.ncbi.nlm.nih.gov/clinvar/ on July 27, 2024), and PDB RCSB PDB 2AHI and 3KZ8.
RNA and DNA sequencing data was deposited at EMBL BioStudies, accession numbers E-MTAB-12734 (bulk RNA-seq), E-MTAB-13904 (single-cell RNA-seq), E-MTAB-14322 (TP53 R175 SGE experiments), E-MTAB-12857 (TP53 exon5-8 SGE genomic DNA sequencing), and E-MTAB-12861 (TP53 exon5-8 SGE cDNA sequencing).

## Research involving human participants, their data, or biological material

Policy information about studies with human participants or human data. See also policy information about sex, gender (identity/presentation), and sexual orientation and race, ethnicity and racism.

| | |
|---|---|
| Reporting on sex and gender | N/A |
| Reporting on race, ethnicity, or other socially relevant groupings | N/A |
| Population characteristics | N/A |
| Recruitment | N/A |
| Ethics oversight | N/A |

Note that full information on the approval of the study protocol must also be provided in the manuscript.

# Field-specific reporting

Please select the one below that is the best fit for your research. If you are not sure, read the appropriate sections before making your selection.

☒ Life sciences ☐ Behavioural & social sciences ☐ Ecological, evolutionary & environmental sciences

For a reference copy of the document with all sections, see nature.com/documents/nr-reporting-summary-flat.pdf

# Life sciences study design

All studies must disclose on these points even when the disclosure is negative.

Sample size

For each experiment, sample size was chosen to obtain sufficient number of experiments and samples to calculate statistical significance.

For SGE experiments, we aimed to investigate all cancer-relevant variants across the parts of the TP53 gene, which encode the DNA-binding domain where >90% of all cancer mutations are located. We therefore included all single-nucleotide variants in exons 5-8 with 12 base pairs of flanking intronic sequence. We further added double and triple nucleotide variants to include all possible amino acid substitutions as well as nonsense and synonymous variants at each codon. Finally, we added all possible single nucleotide insertions and 1-3 base pair deletion,

yielding a sample size of 9,225 variants.

Plasmid preparation and transfection of CRISPR-constructs for generating the mutome library were performed ensuring a minimal coverage of 500 cells/variant. Mutome screens were performed ensuring a minimal coverage of 1000 cells/variant (genomic DNA) or 50 cells/variant (cDNA).

| | |
|---|---|
| Data exclusions | No data could be generated for synonymous mutations at codons for methionine or tryptophan, as no alternative codon is possible. Due to the exon-wise generation of mutome data, some mutations spanning over exon borders couldn't be generated.<br>Variants with lower mean cDNA read counts than 5 were excluded from the analysis. |
| Replication | All experiments were conducted in three replicates, which are all shown in the manuscript and showed excellent correlation. For western blot analysis, representative images of two independent experiments are shown. Data on single cell clones were replicated with 2-10 independent cell clones. |
| Randomization | We performed no randomization in this study because all analyzed TP53 variants were combined with respective negative and positive controls into complex libraries for multiplexed assays of variant effects (MAVE). In these MAVE experiments, variants were introduced into the genome of cells randomly by transfecting cells with the complex library. The resulting cell pools, which contained the variants and controls, were treated and analyzed as pools, and data on individual variants were extracted from these pools. While gDNA libraries and time-point replicates for target regions were grouped together in the same sequencing runs for consistency, the allocation of sequencing runs after grouping was dictated by the availability of gDNA libraries and the timing of experiment readiness, which was essentially random. No additional randomization was performed. |
| Blinding | Blinding was not performed in this study. TP53 variants, along with respective negative and positive controls, were combined into complex libraries for multiplexed assays of variant effects (MAVE). Variants were introduced randomly into the genome via transfection with the pooled library, meaning the identity of the variant in each cell was unknown to the investigator. Consequently, formal blinding was unnecessary as the experimental setup inherently concealed variant identity during analysis. |

# Reporting for specific materials, systems and methods

We require information from authors about some types of materials, experimental systems and methods used in many studies. Here, indicate whether each material, system or method listed is relevant to your study. If you are not sure if a list item applies to your research, read the appropriate section before selecting a response.

## Materials & experimental systems

| n/a | Involved in the study | |
|---|---|---|
| ☐ | ☒ | Antibodies |
| ☐ | ☒ | Eukaryotic cell lines |
| ☒ | ☐ | Palaeontology and archaeology |
| ☐ | ☒ | Animals and other organisms |
| ☒ | ☐ | Clinical data |
| ☒ | ☐ | Dual use research of concern |
| ☒ | ☐ | Plants |

## Methods

| n/a | Involved in the study | |
|---|---|---|
| ☒ | ☐ | ChIP-seq |
| ☐ | ☒ | Flow cytometry |
| ☒ | ☐ | MRI-based neuroimaging |

## Antibodies

| | |
|---|---|
| Antibodies used | p53 (Santa Cruz Biotechnology, sc-126)<br>p21 (Santa Cruz Biotechnology, sc-6246)<br>β-actin (Abcam, ab6276)<br>goat anti-mouse IgG Fc HRP antibody (Invitrogen, A16084)<br>goat anti-rabbit IgG F(ab') HRP antibody (Amersham, NA9340)<br>goat anti-mouse Alexa-488 conjugate (Invitrogen, A-11029) |
| Validation | p53 (Santa Cruz Biotechnology, sc-126): validated by manufacturer by western blot analysis of p53 expression in A549, Daudi and NTERA-2 cell lysates.<br>p21 (Santa Cruz Biotechnology, sc-6246): validated by manufacturer by western blot analysis of p21 Waf1/Cip1 expression in NIH/3T3 cell lysates.<br>β-actin (Abcam, ab6276): validated by manufacturer by western blot analysis of β-actin expression in β-actin HAP1, HeLa, Jurkat, A431, HEK-293, COS-7, NIH/3T3, PC-12 Rat2, CHO, MDBK and MDCK cell lysates.<br>goat anti-mouse IgG Fc HRP antibody (Invitrogen, A16084): validated by manufacturer by western blot analysis on whole cell extracts of K-562 and U-87 MG using anti-SOD1 antibody (Product # MA1-105, 2 µg/mL) and goat anti-mouse IgG Fc HRP antibody<br>goat anti-rabbit IgG F(ab') HRP antibody (Amersham, NA9340): validated by manufacturer by western blot analysis using anti-beta galactosidase antibody (Cappel) and anti-rabbit IgG, HRP F(ab')2 fragment antibody<br>goat anti-mouse Alexa-488 conjugate (Invitrogen, A-11029): validated by manufacturer by immunofluorescence analysis of HeLa cells stained with alpha Tubulin monoclonal Antibody (#A11126) and goat anti-mouse IgG (H+L) Alexa-488 conjugate. |

## Eukaryotic cell lines

Policy information about cell lines and Sex and Gender in Research

| | |
|---|---|
| Cell line source(s) | HCT116: ATCC (CCL-247)<br>NCI-H460: ATCC (HTB-177)<br>HEK 293T: ATCC (CRL-3216)<br>MCF10A: (CRL-10317)<br>LS-123: (CCL-255)<br>MIA-PaCa2: (CRL-1420)<br>H1975: (CRL-5908)<br>PANC-1: (CRL-1469)<br>PC9: ECACC (90071810)<br>AmphoPack-293: Takara Bio Inc. (CVCL_WI47)<br>Normal human diploid fibroblasts (NHDF) and normal human epidermal keratinocytes were obtained from healthy donors |
| Authentication | HCT116 were authenticated by whole genome sequencing.<br>NCI-H460 were authenticated by STR profiling.<br>LS-123, MIA-PaCa2, H1975, PANC-1, PC9, and MCF10A were authenticated by profiling their TP53 mutation status.<br>HEK 293T and AmphoPack-293 were not formally authenticated, but validated functionally by their lentiviral packaging capacity. |
| Mycoplasma contamination | All cell lines were tested negative for mycoplasma contamination prior to and in the course of experiments. |
| Commonly misidentified lines (See ICLAC register) | No commonly misidentified cell lines were used in this study. |

## Animals and other research organisms

Policy information about studies involving animals; ARRIVE guidelines recommended for reporting animal research, and Sex and Gender in Research

| | |
|---|---|
| Laboratory animals | Rag2tm1.1Flv;Il2rgtm1.1Flv male and female mice at a minimal age of eight weeks |
| Wild animals | The study did not involve wild animals. |
| Reporting on sex | This information has not been collected. Both male and female mice were used for this study, as TP53 mutations in cancers are similarly frequent in males and females. |
| Field-collected samples | The study did not involve field-collected samples. |
| Ethics oversight | Mouse experiments were performed in accordance with the German Animal Welfare Law (TierSchG) and received approval from the animal welfare committee of the local authority (Regierungspräsidium Gießen). |

Note that full information on the approval of the study protocol must also be provided in the manuscript.

## Plants

| | |
|---|---|
| Seed stocks | N/A |
| Novel plant genotypes | N/A |
| Authentication | N/A |

# Flow Cytometry

## Plots

Confirm that:

☒ The axis labels state the marker and fluorochrome used (e.g. CD4-FITC).

☒ The axis scales are clearly visible. Include numbers along axes only for bottom left plot of group (a 'group' is an analysis of identical markers).

☒ All plots are contour plots with outliers or pseudocolor plots.

☒ A numerical value for number of cells or percentage (with statistics) is provided.

## Methodology

| | |
|---|---|
| Sample preparation | As described in the methods section, cells and media supernatants were collected, pelleted, and resuspended in Annexin-V-APC conjugate (MabTag, AnxA100) diluted in Annexin V binding buffer (BD Biosciences, 556454) according to the manufacturer's protocol. The suspension was incubated in the dark for 20 minutes at RT, washed in Annexin V binding buffer and analyzed by flow cytometry on a BD LSR II Flow Cytometer or a BD Accuri C6 Plus Flow Cytometer. Sorting of Annexin V stained cells was performed using a Beckman Coulter MoFlo Astrios sorter . |
| Instrument | BD LSR II Flow Cytometer, BD Accuri C6 Plus Flow Cytometer, Beckman Coulter MoFlo Astrios sorter |
| Software | BD FACSDiva, BD Accuri C6 Plus (v1.0.23.1), Summit (v6.3.1), FlowJo (v10.8.1) |
| Cell population abundance | does not apply |
| Gating strategy | 1.) SSC-H vs. FSC-H gating to exclude debris<br>2.) FSC-H vs. FSC-A gating to exclude doublets<br>3.) APC-A (annexin-V) vs. FITC-A (GFP) gating to quantify / sort GFP negative and annexin-V positive / annexin-V negative cells.<br>Unrecombined, completely recombined and unstained cells were used as controls to set up the gating.<br>See Supplementary Figure 10. |

☒ Tick this box to confirm that a figure exemplifying the gating strategy is provided in the Supplementary Information.
