# Supplementary material for: Deep CRISPR mutagenesis characterizes the functional diversity of TP53 mutations
Source: Nat Genet. 2025 Jan 7;57(1):140–53. doi: 10.1038/s41588-024-02039-4 (PMC11735402; doi:10.1038/s41588-024-02039-4)

Figure 1c

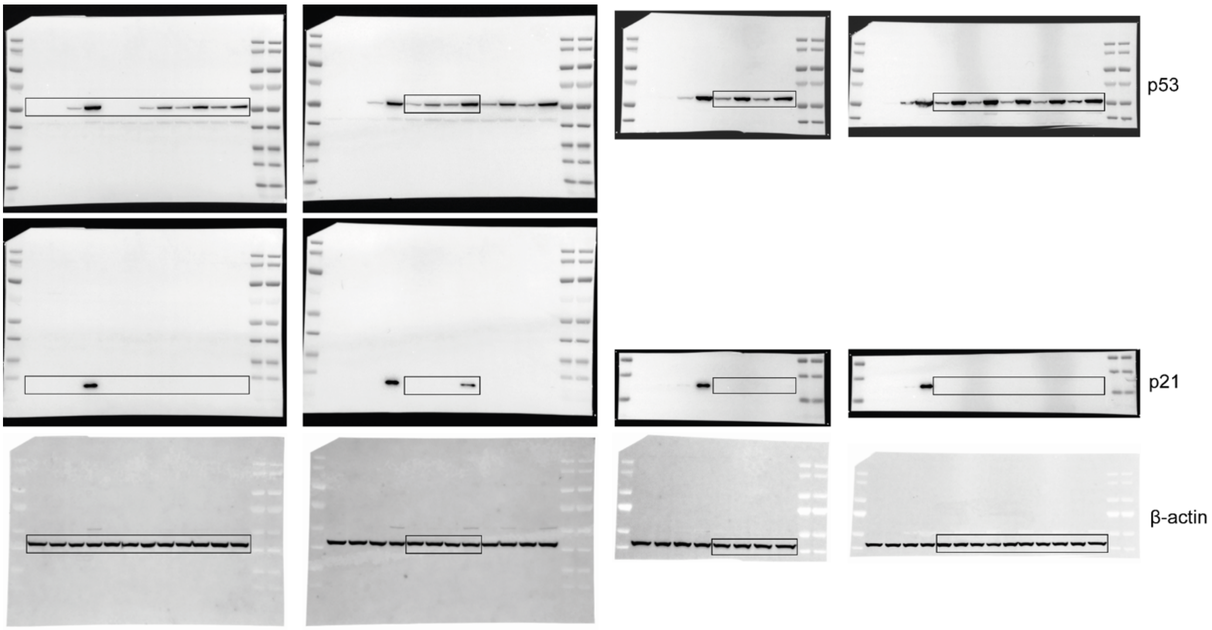

Figure 7h

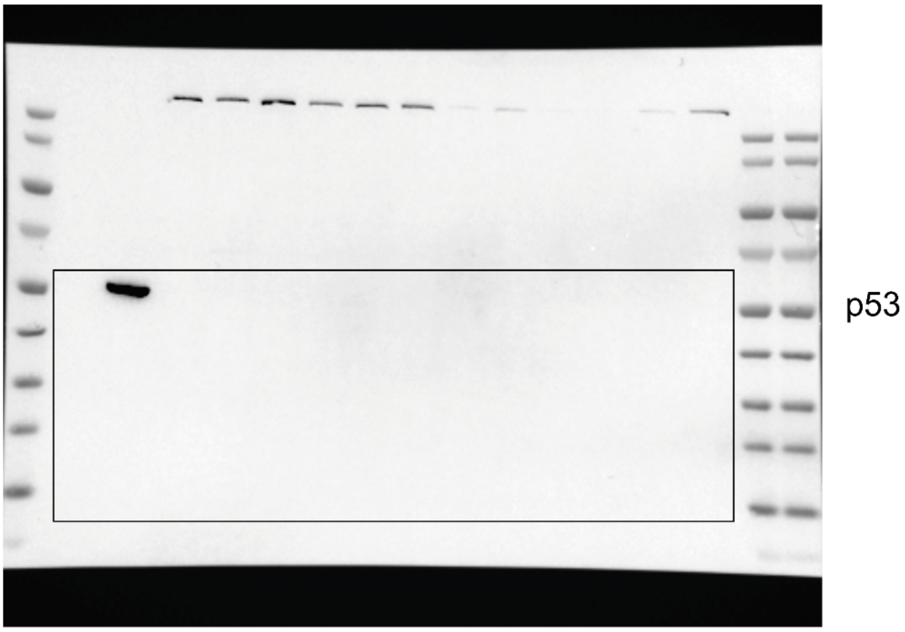

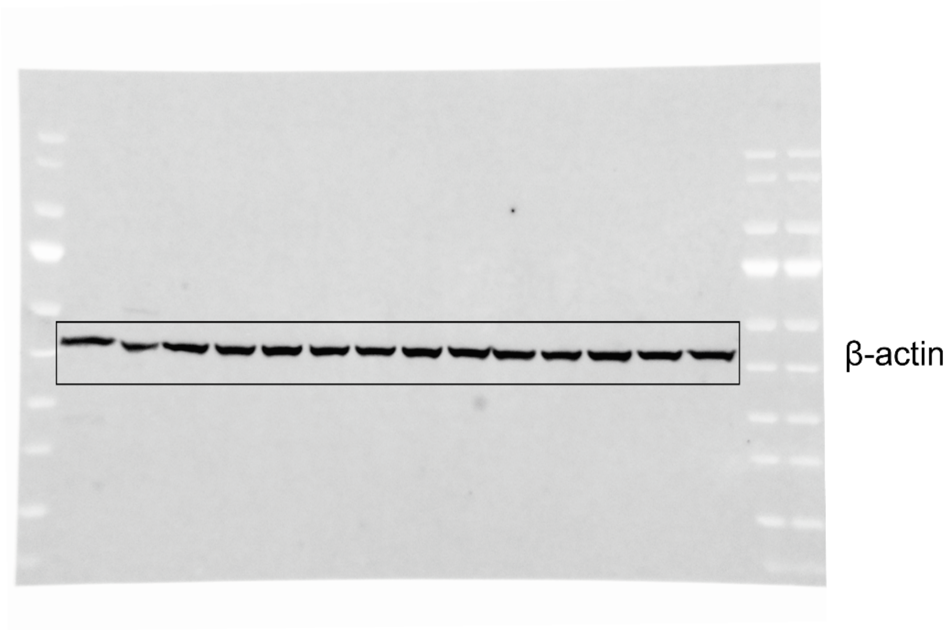

Figure 8d

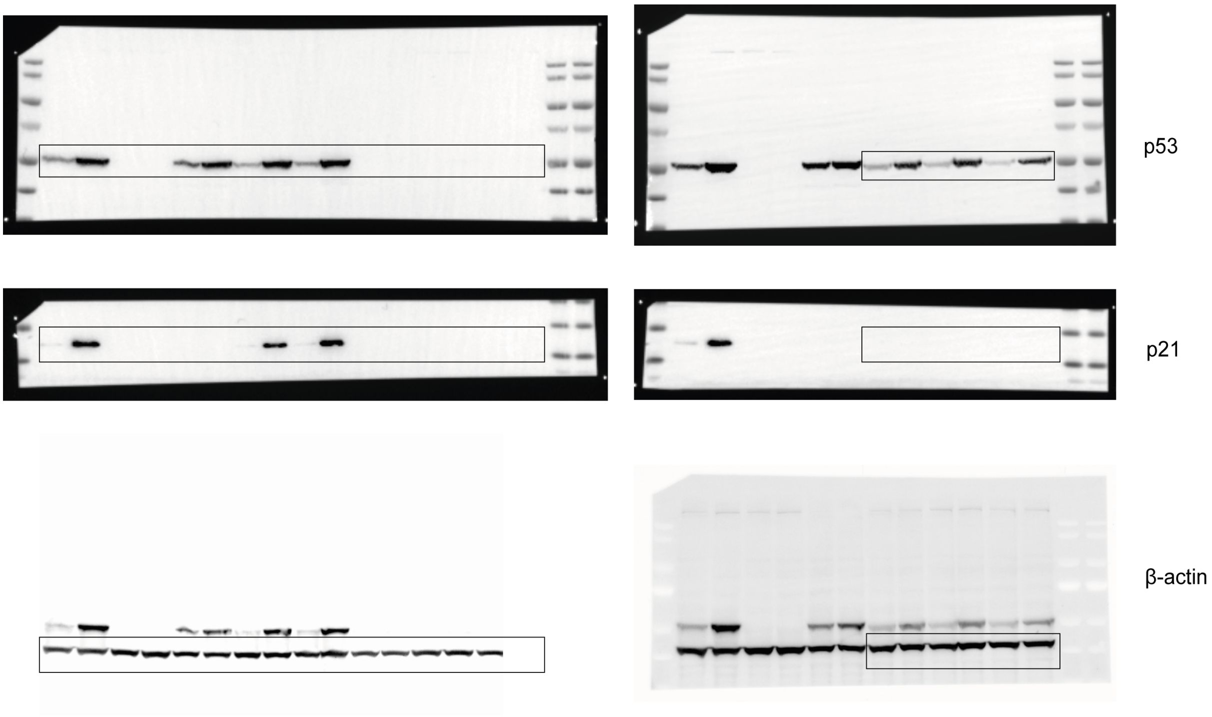

Figure 8e

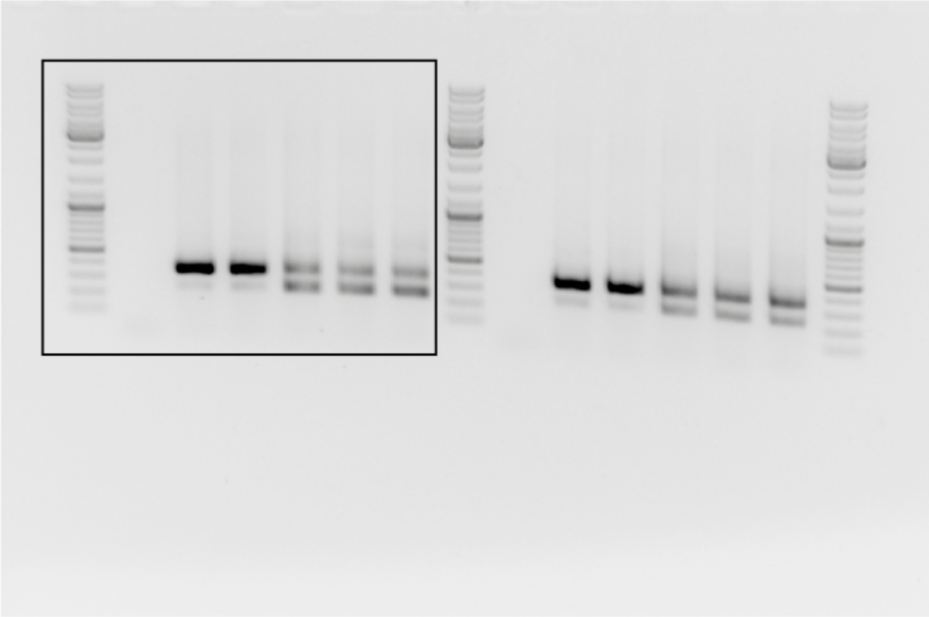

Figure 8g

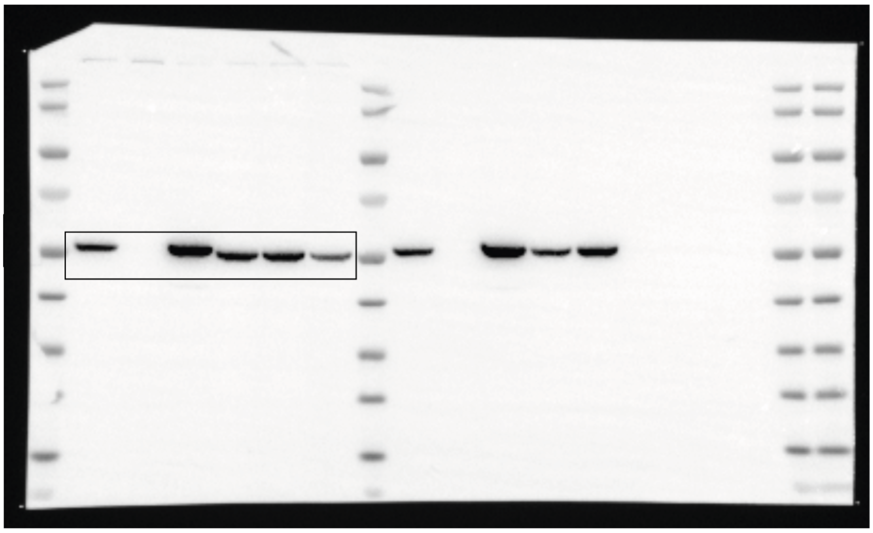

p53

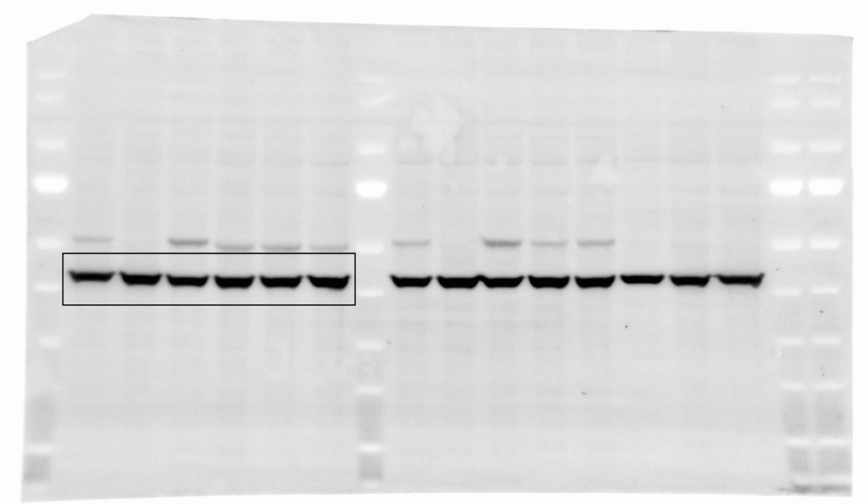

β-actin

Figure 8h

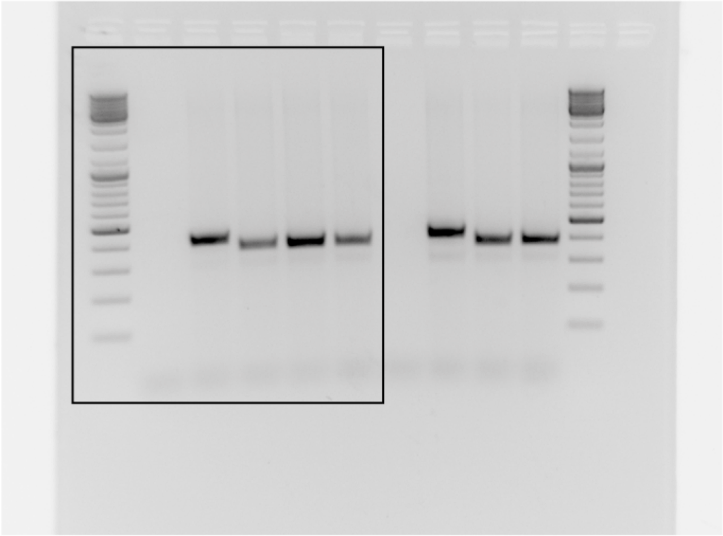

Extended Data Figure 1d

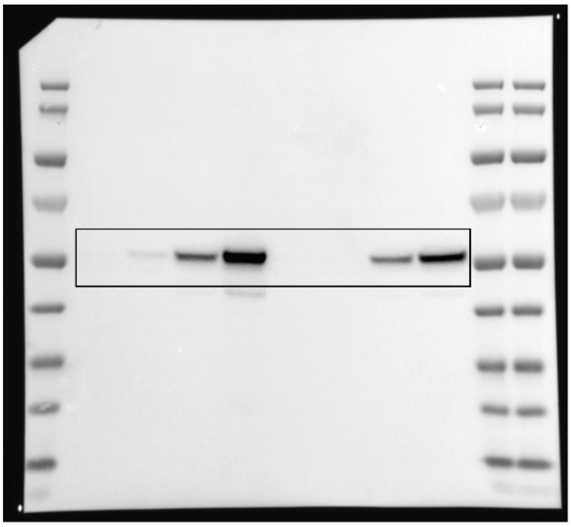

p53

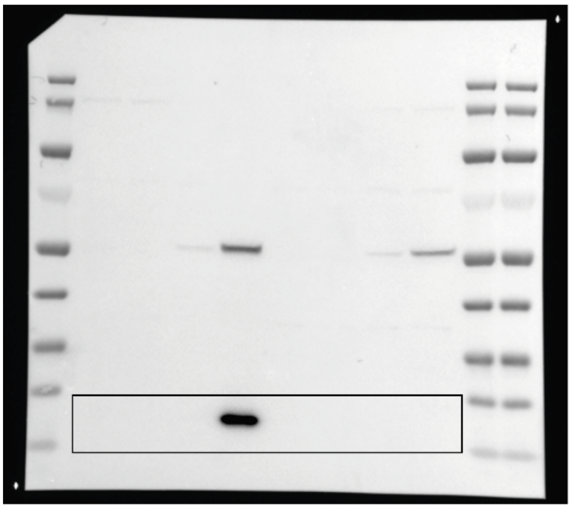

p21

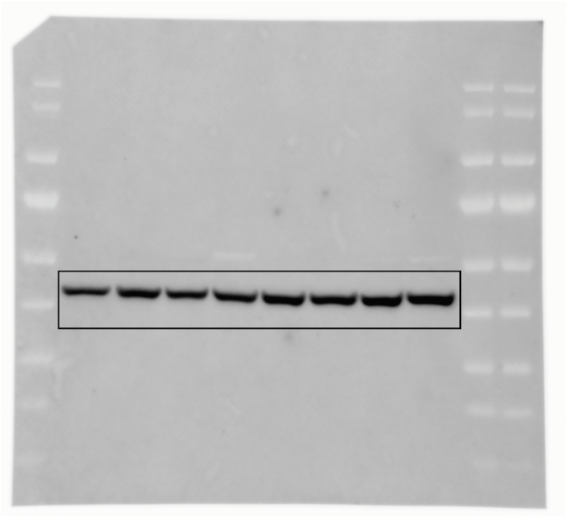

β-actin

Extended Data Figure 1e

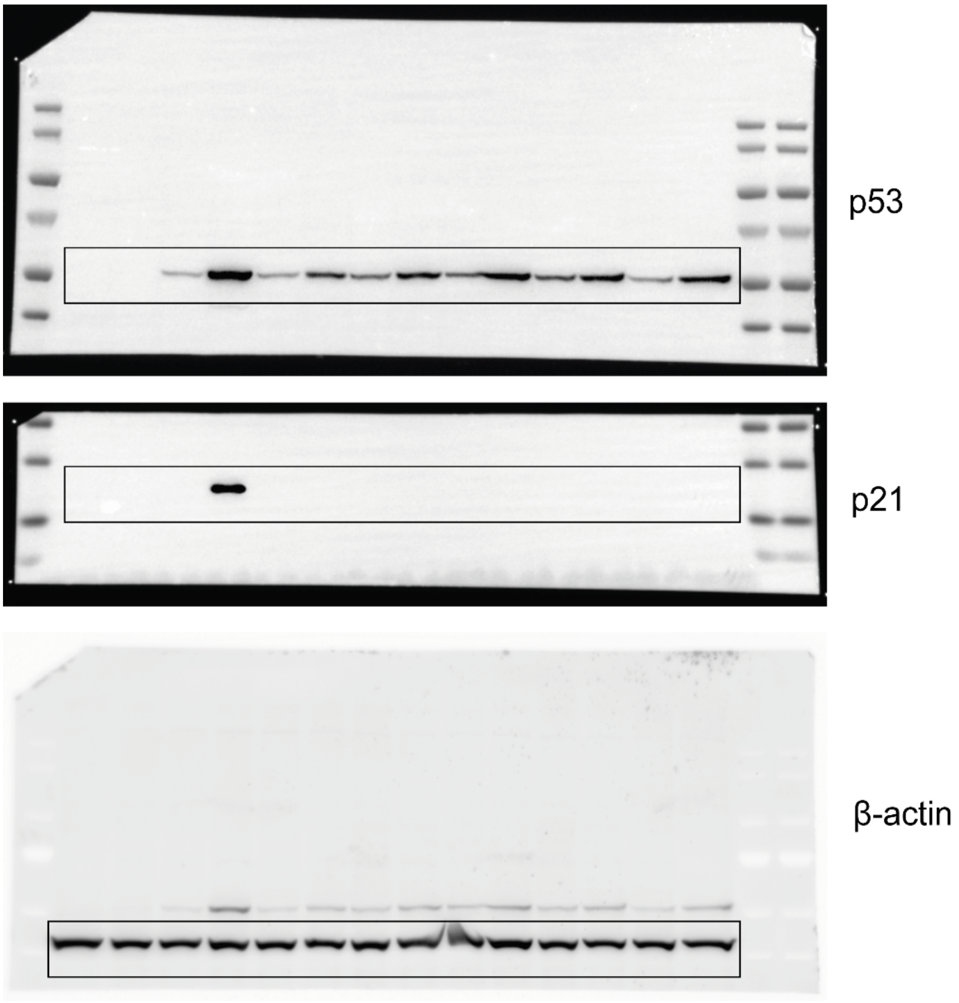

Extended Data Figure 1h

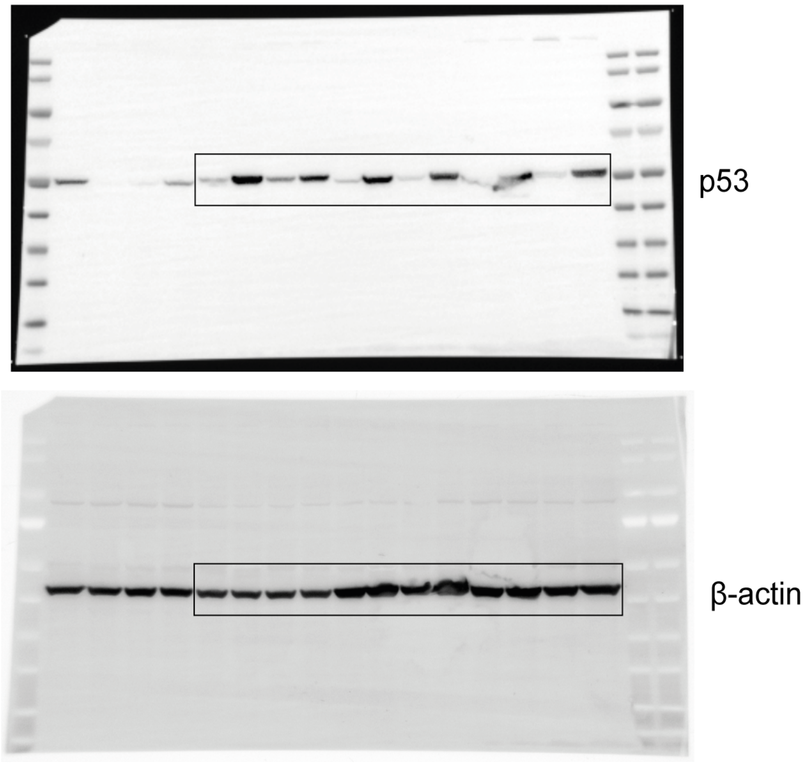

Extended Data Figure 1i

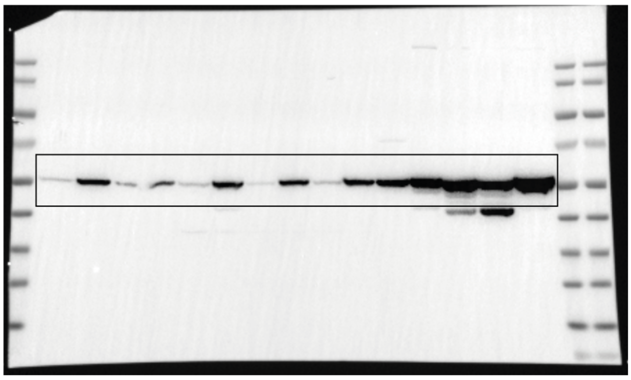

p53

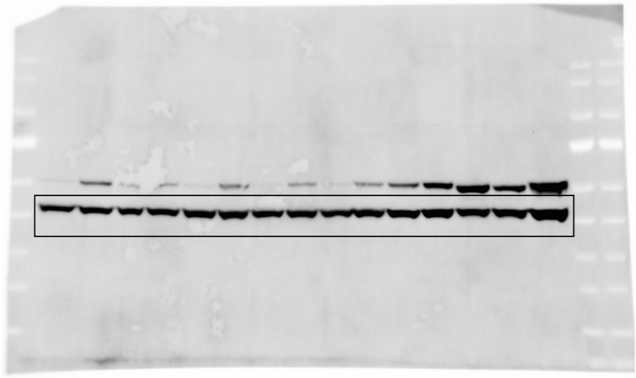

β-actin

Extended Data Figure 3a

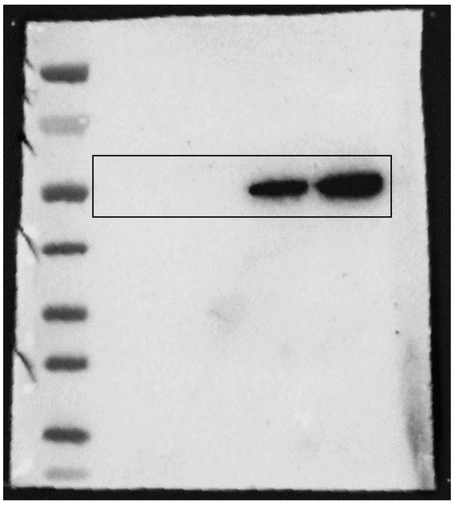

p53

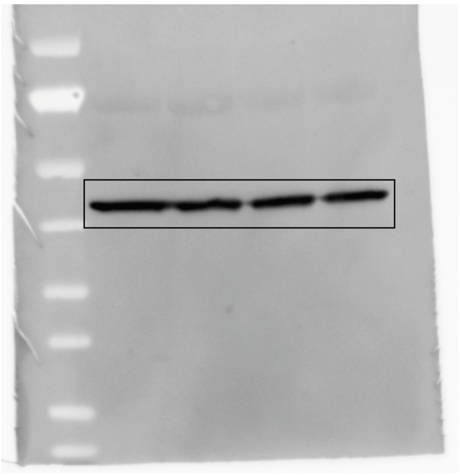

β-actin

Extended Data Figure 3e

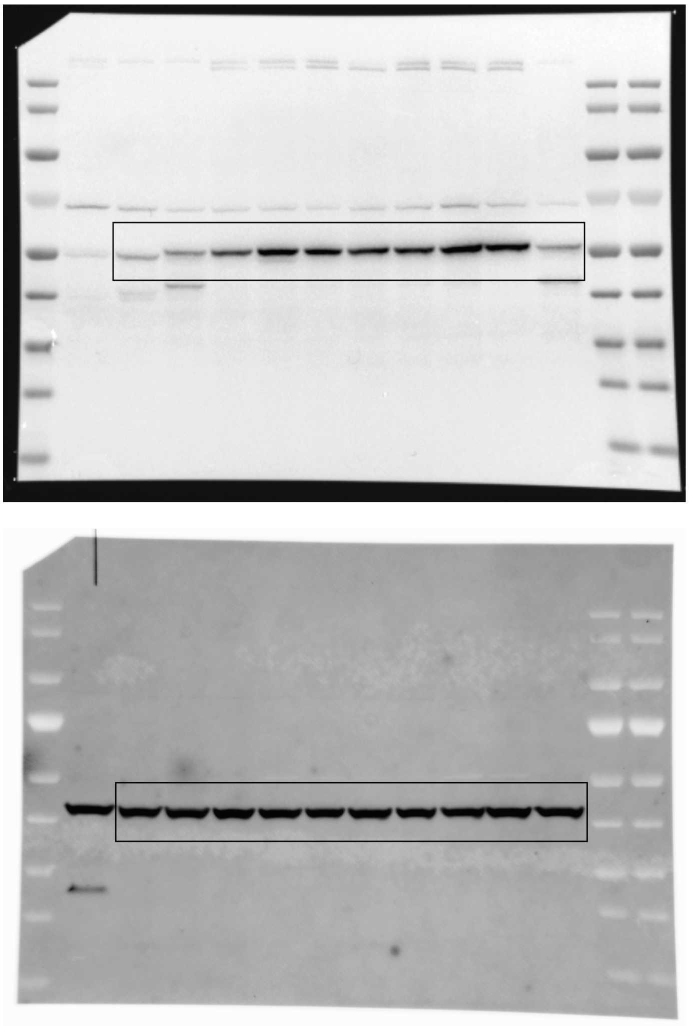

p53

β-actin

Extended Data Figure 3h

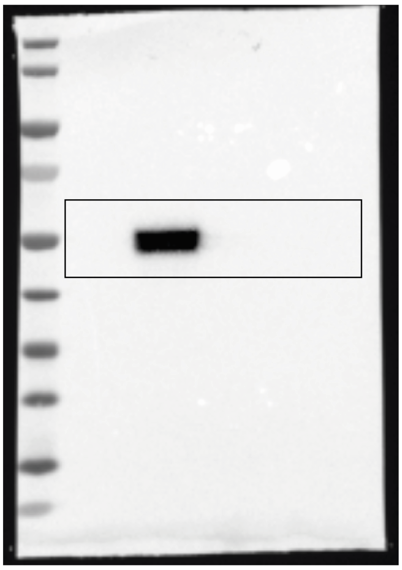

p53

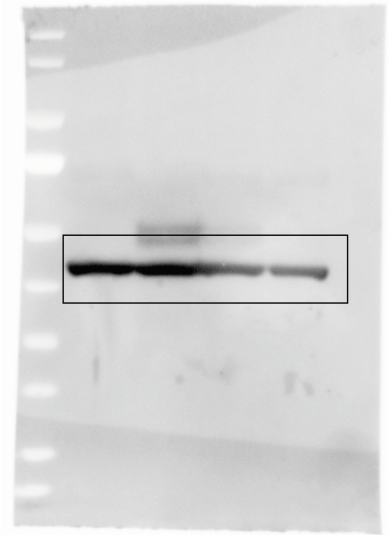

β-actin

Extended Data Figure 3j

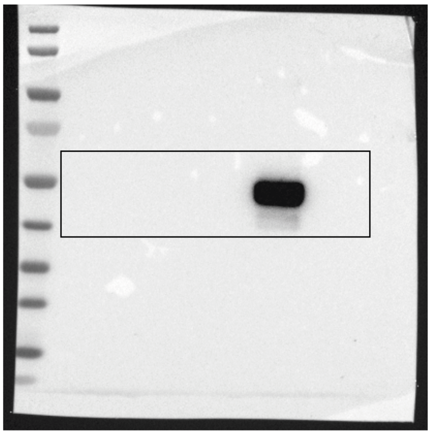

p53

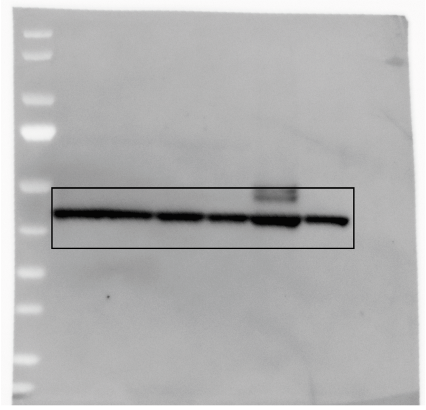

β-actin

Extended Data Figure 3l

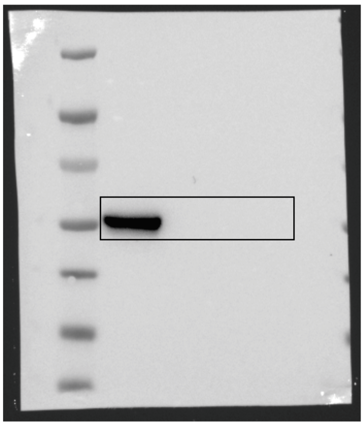

p53

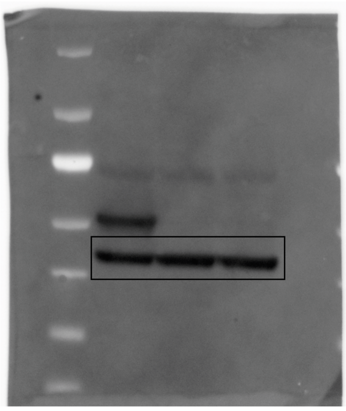

β-actin

Extended Data Figure 4d

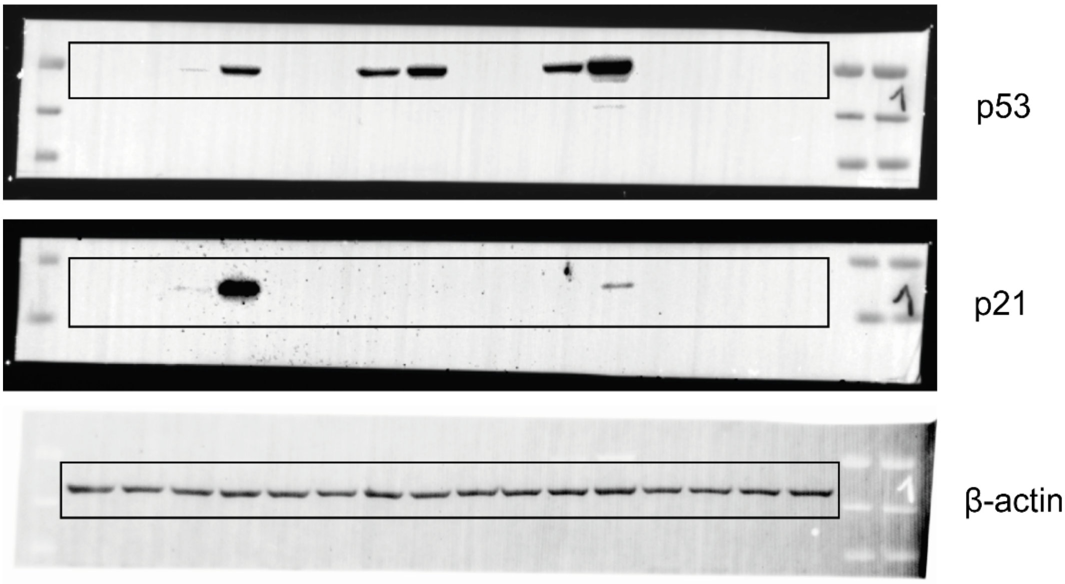

Extended Data Figure 10d

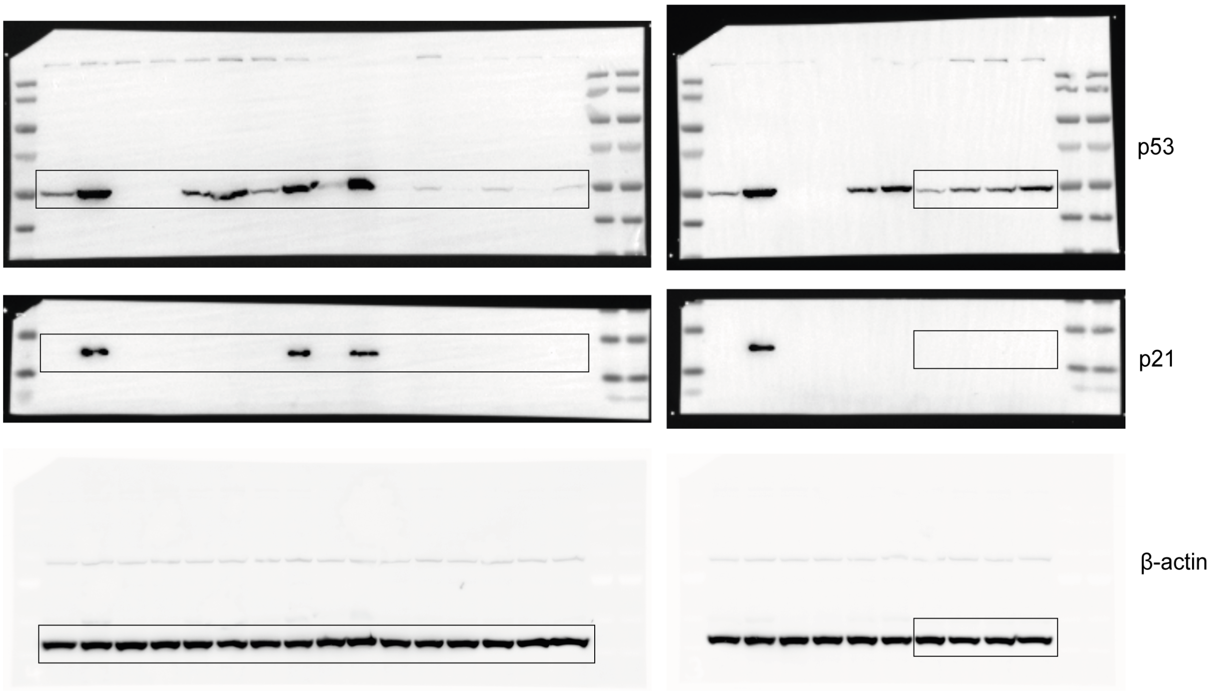

Extended Data Figure 10e

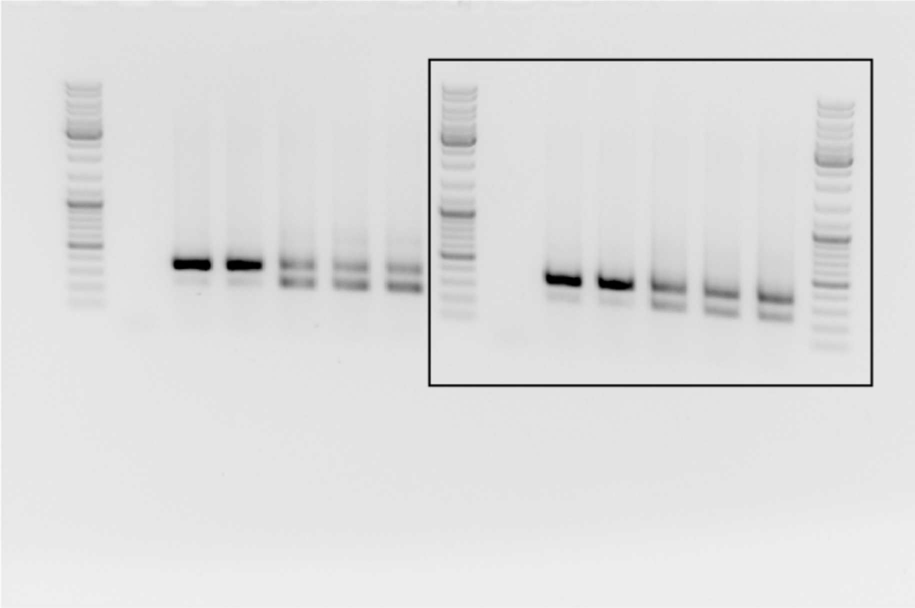

Extended Data Figure 10g

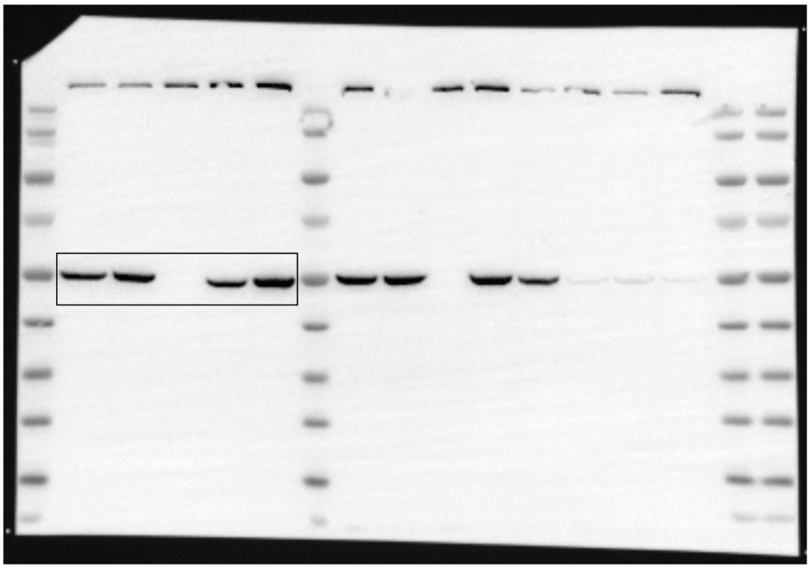

p53

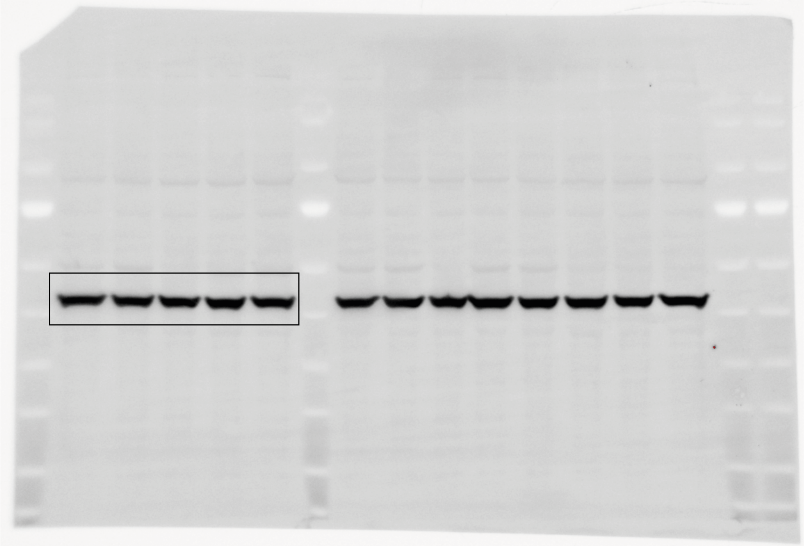

β-actin

Extended Data Figure 10h

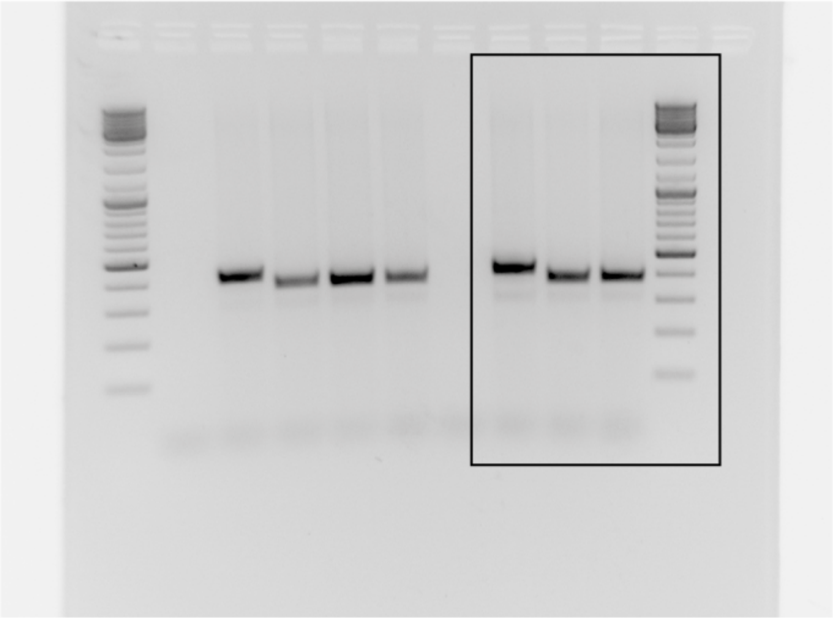

Supplement: Supplementary file 15 — Unprocessed western blots and/or gels for Figs. 1, 7 and 8 and Extended Data Figs. 1, 3, 4 and 10. [file 41588_2024_2039_MOESM15_ESM.pdf]
